# The JAK/STAT signaling pathway: from bench to clinic

**DOI:** 10.1038/s41392-021-00791-1

**Published:** 2021-11-26

**Authors:** Xiaoyi Hu, Jing li, Maorong Fu, Xia Zhao, Wei Wang

**Affiliations:** 1 State Key Laboratory of Biotherapy and Cancer Center, West China Hospital, Sichuan University, and Collaborative Innovation Center for Biotherapy Chengdu, 610041 Sichuan, P. R. China; 2 Department of Gynecology and Obstetrics, Development and Related Disease of Women and Children Key Laboratory of Sichuan Province, Key Laboratory of Birth Defects and Related Diseases of Women and Children, Ministry of Education, West China Second Hospital, Sichuan University, 610041 Chengdu, P. R. China

**Keywords:** Immunopathogenesis, Molecular medicine, Cancer, Molecular biology

## Abstract

The Janus kinase/signal transducer and activator of transcription (JAK/STAT) signaling pathway was discovered more than a quarter-century ago. As a fulcrum of many vital cellular processes, the JAK/STAT pathway constitutes a rapid membrane-to-nucleus signaling module and induces the expression of various critical mediators of cancer and inflammation. Growing evidence suggests that dysregulation of the JAK/STAT pathway is associated with various cancers and autoimmune diseases. In this review, we discuss the current knowledge about the composition, activation, and regulation of the JAK/STAT pathway. Moreover, we highlight the role of the JAK/STAT pathway and its inhibitors in various diseases.

## Introduction

The Janus kinase/signal transducer and activator of transcription (JAK/STAT) signaling pathway is regarded as one of the central communication nodes in the cell function. More than 50 cytokines and growth factors have been identified in the JAK/STAT signaling pathway, such as hormones, interferons (IFN), interleukins (ILs), and colony-stimulating factors.^1^ JAK/STAT-mediated downstream events vary and include hematopoiesis, immune fitness, tissue repair, inflammation, apoptosis, and adipogenesis.^2^ Loss or mutation of JAK/STAT components is related to many diseases in humans. JAKs are noncovalently associated with cytokine receptors, mediate tyrosine phosphorylation of receptors, and recruit one or more STAT proteins. Tyrosine-phosphorylated STATs dimerize and are then transported into the nucleus through the nuclear membrane to regulate specific genes. Although STATs can be activated by partially overlapping cytokines, different STATs have nonredundant biological effects.^3^

The JAK/STAT signaling pathway has profoundly influenced recent understanding attained of human health and disease. Many papers have reported the importance of this pathway in malignancies and autoimmune diseases.^4–9^ Thus, inhibiting the JAK/STAT pathway is promising for treating various diseases. Currently, many JAK inhibitors have achieved efficacy in many clinical settings, and more medications are currently being studied.^10^ In this review, we aim to provide updated and comprehensive views of the JAK/STAT signaling pathway at the cellular, molecular, and genomic levels, and elucidate the relationship between JAK/STAT pathway components and human diseases. Finally, we focus on the current market-approved and preclinical medications designed to target this pathway.

## Discovery of the JAK/STAT signaling pathway

The JAK/STAT signaling pathway was first discovered when studying how IFNs lead to the activation of a transcription factor.^11^ In 1990, the transcriptional activator interferon-stimulated gene factor 3 (ISGF3), a transcription factor that responds to IFN-α, was discovered to be composed of multiple interacting polypeptide chains (48, 84, 91, and 113 kDa).^11^ In 1992, Fu reported that 113, 91, and 84 kDa (ISGF3α) proteins of ISGF3 contain conserved SH2 and SH3 domains. Moreover, a specific IFN-α-induced cytoplasmic tyrosine kinase can phosphorylate and activate ISGF3α. Thus, Fu proposed a direct effector model for signal transduction induced by IFN-α, which revealed the signal transduction mode of the JAK/STAT signaling pathway.^12,13^ Later studies have identified that ISGF3 is comprised of STAT1, STAT2, and IRF9.^14^ Since then, STAT3, STAT4, STAT5a, STAT5b, and STAT6 were found in several laboratories during 1993–1995.^15–17^

The discovery of JAKs happened in 1989–1994. In 1989, Wilks et al. identified that a tyrosine kinase has a recognizable kinase domain and a pseudokinase domain. In 1991, they found a second tyrosine kinase with this feature. Wilks called them JAK1 and JAK2.^18,19^ The other two JAKs, tyrosine kinase 2 (TYK2) and JAK3, were identified in 1990 and 1994.^20^ The connection between JAKs and STATs began in 1992 when Velazquez et al. discovered that TYK2 is an essential protein in the IFN-α/β signaling pathway.^21^ Later, Müller et al. found that IFN-dependent signaling required JAKs to phosphorylate STATs.^22^ Thus, in the late 1980s to early 1990s, the components and outlines of the JAK/STAT signaling pathway were completed. Research on more proteins and functions of the JAK/STAT pathway has continued to the present, making the JAK/STAT landscape more abundant.

## Composition of the JAK/STAT pathway

The JAK/STAT signaling pathway is evolutionarily conserved. It is composed of ligand-receptor complexes, JAKs, and STATs. There are 4 members in the JAK family: JAK1, JAK2, JAK3, and TYK2. The STAT family comprises seven members: STAT1, STAT2, STAT3, STAT4, STAT5a, STAT5b, and STAT6. We introduce them by family.

### The JAK family: JAK1, JAK2, TYK2, and JAK3

**Fig. 1:**
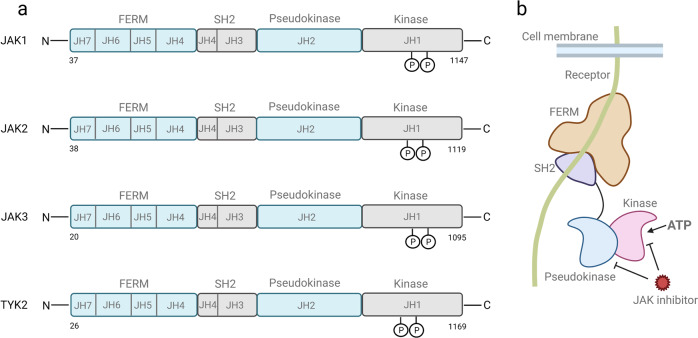
Structure of JAKs. **a** Structure and conserved phosphorylation sites of the JAK family. The JAK family has four main members: JAK1, JAK2, JAK3, and TYK2. Each is composed of seven homology domains (JH), of which JH1 constitutes the kinase domain; JH2 constitutes the pseudokinase domain; a part of JH3 and JH4 together constitute the SH2 domain; and the FERM domain is composed of the JH5, JH6, and part of the JH4 domains. The conserved tyrosine phosphorylation sites in JAK1 are Y^1038^/Y^1039^; the conserved tyrosine phosphorylation sites in JAK2 are Y^1007^/Y^1008^; the conserved tyrosine phosphorylation sites in JAK3 are Y^980^/Y^981^; the conserved tyrosine phosphorylation sites in Tyk2 are Y^1054^/Y^1055^. **b** Structure of JAKs and targeting sites of JAK inhibitors. Created with BioRender.com

JAK family consists of non-receptor tyrosine protein kinases (Fig. 1). When cytokines bind to their receptors, JAK tyrosine kinases are activated and transmit regulatory signals. The JAK family has four main members, JAK1, JAK2, JAK3, and TYK2. JAK3 is only expressed in the bone marrow and lymphatic system, as well as endothelial cells and vascular smooth muscle cells,^23,24^ other members are expressed in almost all tissues.^19,20,25–29^ JAKs have seven homology domains (the JAK homology domain, JH). Starting from the carboxyl terminus, JH1 is the first JH, known as the kinase domain, and is composed of approximately 250 amino acid residues. JH1 encodes a kinase protein that constitutes the kinase structure domain that phosphorylates a substrate; JH2 is a PK domain. JH2 is structurally similar to the kinase domain but has no kinase activity. Its main function is to regulate the activity of the kinase domain. The pseudokinase domain participates in the interaction of JAK and STAT, and the PK domain can also inhibit Tyr kinase activity by binding to the kinase domain; JH3 with one-half of JH4 constitutes the Src-homology 2(SH2) domain, the combination of one-half of JH4, JH5, JH6, and JH7 constitutes the FERM domain, and the SH2 and FERM domains mainly regulate the binding of JAK and cytokine-receptor membrane-proximal box1/2 regions.^19,25,30–32^

### JAK1

Y^1038^/Y^1039^ in JAK1 is a conserved tyrosine that constitutes a vital part of the activation loop. The phosphorylation of a double tyrosine in the SH1 domain of each JAK results in a more favorable conformation for substrate binding.^33^ JAK1 is widely expressed in tissues and can phosphorylate all STATs.^4^ JAK1 is phosphorylated by four cytokine-receptor families: (1) Cytokine receptors with the γc receptor subunit, IL-2 receptor, IL-4 receptor, IL-7 receptor, IL-9 receptor, and IL-15 receptor; (2) class II cytokine receptors include the IFNα/β receptor, IFN-γ receptor, and IL-10 family cytokine receptors; and (3) receptors with a gp130 subunit, including the IL-6 receptor, IL-11 receptor, ciliary neurotrophic factor (CNTF) receptor, oncostatin M (OSM) receptor, leukemia inhibitory factor (LIF) receptor, and cardiotrophin-1 (CT-1) receptor.^34^ JAK1 can promote body haematopoietic function after being activated by IL-3, IL-5, IL-7, granulocyte–macrophage colony-stimulating factor (GM-CSF), or granulocyte colony-stimulating factor (G-CSF).^35^ JAK1−/− mice are perinatal dead and exhibit neurological disease and severe lymphocyte damage caused by deficient of LIF and IL-7 signal, respectively.^34^

### JAK2

The conserved tyrosine sites in JAK2 are Y^1007^ and Y^1008^.^33^ Similar to JAK1, JAK2 can also be phosphorylated by members of the gp130 receptor family and class II cytokine-receptor family. It also participates in the signal transduction of the IL-3 receptor family (IL-3R, IL-5R, and GM-CSF receptor), and single-chain receptors (such as erythropoietin receptor (EPO), growth hormone (GH) receptor, prolactin receptor, and thrombopoietin (TPO) receptor).^36^ JAK2-knockout mice die at approximately 12 days of gestation primarily due to the impaired hematopoietic function mediated by EPO. Therefore, the embryonic lethality of JAK2-knockout mice and EPO-knockout mice is very similar.^37,38^ JAK2-knockout mice exhibit specific defects in IFN-γ-related biological responses, but they do not respond to IFN-α or IFN-β.

### JAK3

Y^980^/Y^98^ in JAK3 are the conserved phosphorylation sites.^33^ JAK3 is mainly involved in the signal transduction of the IL-2 receptor, IL-4 receptor, IL-7 receptor, IL-9 receptor, IL-15 receptor, and IL-21 receptor. These receptors are γC receptors with the γ receptor chain.^39^ JAK3-knockout mice are defective in lymphocyte production due to the lack of γC signaling. These mice are very likely to have severe combined immunodeficiency, but JAK3-knockout mice can still survive in the absence of specific pathogens.^40,41^ IL-2, IL-4, and IL-7 transmit growth signals through JAK3, and autoreactive T cells in JAK3-deficient mice are permanently activated. Lack of JAK3 may lead to autosomal recessive combined immunodeficiency, indicating that JAK3 plays an important regulatory role in the negative selection of T cells and the maintenance of the normal phenotype and function of peripheral T cells.^42^

### TYK2

Y^1054^/Y^1055^ in Tyk2 are conservative phosphorylation sites.^33^ Tyk2 was the first discovered member of the JAK family and was originally found to be able to transmit IFN-α/β signals.^43^ Later, it was discovered that Tyk2 is also involved in IL-6,^44^ IL-10,^45^ IL-12,^46^ IL-13,^47^ and IL-23 signaling.^48^ Interestingly, Tyk2-knockout mice do not completely lose cytokine signaling and exhibit partial defects in IFN-α, IFN-β, and IL-12 signal transduction.^49^ Tyk2-defective mice show an insufficient response to a small amount of IFN-α, and increasing the amount of IFN-α can restore signal transduction. Thus, Tyk2 does not seem necessary for type I interferon signal transduction.^50^ Moreover, Tyk2 regulates the balance of Th1 and Th2 cells in mice and regulates the allergic reaction mediated by Th2 cells.^51^ The symptoms of Tyk2 deficiency in human are somewhat different from those in mice. In the clinic, patients with high immunoglobulin E syndrome have defective signal transduction of IFN-α, IL-12, IL-6, and IL-10, which can be alleviated by treating with Tyk2 gene transduction therapy. In patients with Tyk2 deficiency, the phosphorylation of STAT cannot be detected even when they are treated with high concentrations of IFN-α. Tyk2-defective humans develop severe allergic phenotypes due to IFN-mediated loss of antimicrobial capacity. These studies have shown that Tyk2 plays a necessary role in human innate and acquired immunity (Table 1).^50^

**Table 1:** Activated JAK family-related cytokine receptors and JAK−/− mouse phenotype

Janus kinases	Cytokine-receptor signaling	Phenotype
JAK1	(1) Cytokine of the γc receptor subunit (IL-2R, IL-4R, IL-7R, IL-9R, and IL-15R) IL-21R (2) Class II cytokine receptor (IFNα/βR, IFN-R, and IL-10 family cytokine receptor) (3) Receptor with gp130 subunit: (IL-6R, IL-11R, CNTF-R, OSM-R, LIF-R, CT-1 receptor)	(1) Death during the perinatal period. (2) Lymphocyte damage.
JAK2	(1) Gp130 receptor family (2) The class II cytokine-receptor family (3) IL-3 receptor family (IL-3R, IL-5R, and GM-CSF receptor) (4) Single-chain receptors (GH-R, EPO-R, TPO-R, PRL-R)	(1) Deficiency of primordial red blood cells and hepatic red blood cells leads to embryonic death. (2) There are defects in IFN-related biological reactions.
JAK3	All of γC receptors: (IL-2R, IL-4R, IL-7R, IL-9R, IL-15R, IL-21R)	(1) Insufficient γC signal leads to defective lymphocyte production, which may cause SCID. (2) Regulate the negative selection of T cells and maintain the phenotype and function of peripheral T cells.
Tyk2	IFN-α/β, IL-6R family, IL-10R family, IL-12R, Il-13R, IL-23R	(1) There are defects in the signal conduction of IFN-Is and IL-12. (2) Decreased T-cell response, unable to clear the virus.

*JAK* Janus kinase, *TYK2* tyrosine kinase 2, *IFN* interferon, *CNTF-R* ciliary neurotrophic factor receptor, *OSM-R* oncostatin M receptor, *LIF-R* leukemia inhibitory factor receptor, *CT-1* cardiotrophin-1, *GM-CSF-R* granulocyte–macrophage colony-stimulating factor receptor, *GH-R* growth hormone receptor, *EPO-R* erythropoietin receptor, *TPO-R* thrombopoietin receptor, *PRL-R* prolactin receptor, *SCID* severe combined immunodeficiency

### The STAT family: STAT1, STAT2, STAT3, STAT4, STAT5a, STAT5b, and STAT6

**Fig. 2:**
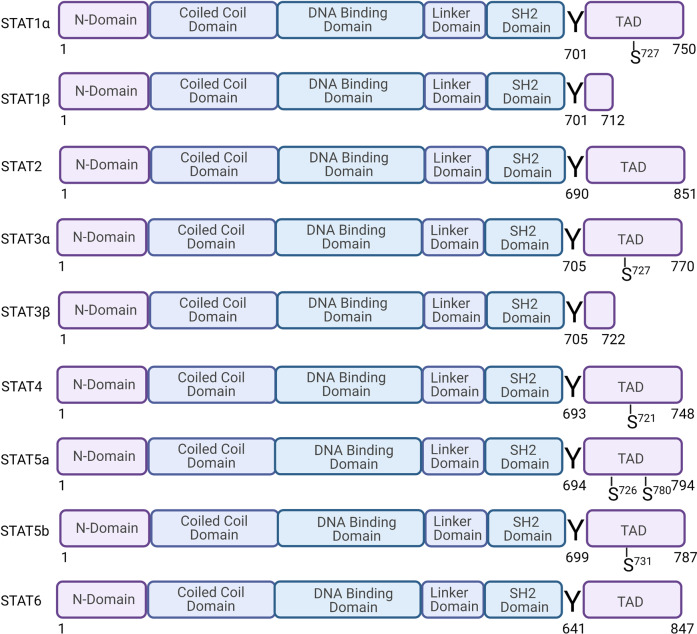
Structure and phosphorylation sites of the STAT protein family. The signal transduction and activator of transcription (STAT) family have six members: STAT1 (STAT1 has two splicing isoforms, STAT1α and STAT1β), STAT2, STAT3 (STAT3 has two splicing isoforms, STAT3α and STAT3β), STAT4, STAT5a, STAT5b, and STAT6. STATs are composed of 750–900 amino acids. From the N-terminus to the C-terminus, the domains are the nitrogen-terminal domain, coiled-coil domain, DNA-binding domain, junction domain, SH2 domain, and transcription-activation structure. "Y" represents a tyrosine phosphorylation site, and "S" represents a serine phosphorylation site. Created with BioRender.com

STAT family is composed of STAT1, STAT2, STAT3, STAT4, STAT5a, STAT5b, and STAT6 (Fig. 2). STAT family members consist of 750–900 amino acids. From the N-terminus to the C-terminus, there are the N-terminal domain and coil, helix domain, DNA-binding domain, connection domain, SH2 domain, and transcription-activation domain. Six domains regulate different functions of STAT.^12,15,17,52–55^ (1) The N-terminal domain promotes the formation of STAT dimers, which enables their subsequent binding with transcription factors. Studies have also shown that the N-terminus can also promote the interaction of STAT and transcription co-activators, the PIAS family, and receptors and regulate nuclear translocation.^56–59^ (2) The coiled-coil domain is composed of a potentially dynamic four-helix bundle. This domain is related to regulatory proteins and participates in the control of nuclear import and export processes. It can interact with p48/IRF9, Nmi, c-Jun, StlP, etc.^60–66^ (3) The linking domain, as the name implies, structurally connects the DNA-binding domain to the SH2 domain. It is involved in the transcriptional regulation of STAT1.^63,67^ (4) The DNA-binding domain can recognize and bind to the DNA sequence in the regulatory region of the target gene. It also participates in the regulation of nuclear import and export. (5) The SH2 domain of STAT is very different from other SH2 domains, but this domain is very conserved in the STAT family.^68^ The primary function of SH2 is to recognize phosphotyrosine motifs of cytokine receptors. Moreover, the SH2 domain cooperates with activated JAK to drive the SH2 domain of STAT to interact with the tail of another STAT monomer after phosphorylation to form a homodimer or heterodimer.^69–72^ (6) The transcriptional activation domain is critical for DNA transcription elements and the recruitment of co-activators through a conserved serine phosphorylation site and regulating the transcription. STAT4, STAT5, and STAT6 can be used as targets for ubiquitin-dependent destruction, while STAT1, STAT2, and STAT3 are more stable, indicating that the transcriptional active region also regulates protein stability.

#### STAT1

STAT1 has two splice isomers. One is STAT1α, with a size of 91 kD. STAT1α, similar to other STATs, has a complete transcription-activation domain. The amino acid positions 701 and 727 are the two phosphorylation sites, while most of the transcriptional activation region in the protein form of STAT1β is missing. The size of STAT1β is 84 kD, and STAT1β has only one phosphorylation site, at the amino acid 701.^73^ Functionally, STAT1β can respond to IFN-I ligands, but its response to IFN-γ is defective and may have an antagonistic effect on STAT1α.^74^ STAT1 is mainly activated by IFN. In addition, other cytokines, including IL-2, IL-6, platelet-derived growth factor, epidermal growth factor (EGF), hepatocyte growth factor, tumor necrosis factor (TNF), and angiotensin II can also activate STAT1. The biological function of STAT1 includes the following aspects: (1) Inhibits cell growth. STAT1 can inhibit cell growth by regulating the expression of cell cycle-related genes, such as promoting the expression of the Cyclin-dependent kinase inhibitors P21 and P27 or inhibiting the expression of c-myc. STAT1 can also control cell growth by inhibiting the expression of cyclin.^75,76^ (2) Regulates cell differentiation. Phosphorylation of STAT1 can regulate the differentiation of human granulocytes and osteoblasts.^77,78^ (3) Promotes cell apoptosis. The expression of STAT1 can promote the expression of a series of apoptosis proteins, which is the leading way for STAT1 to promote apoptosis. For example, STAT1 induces the formation of apoptotic protein caspase1, 3, and 11 precursors, and interacts with the p53 protein.^79–81^ Moreover, STAT1 can also induce Fas, Bcl-2, and Bcl-X gene expression.^82,83^ (4) Inhibits tumor occurrence. In STAT1/p53-knockout mice, the spontaneous or induced tumor formation rate was higher than that in mice in which only p53 was knocked out, and the antitumor activity of IFN-α disappeared. Simultaneously, in human tumors, such as breast cancer and Wilms tumor, the expression of STAT1 is associated with a better prognosis.^84^ However, some studies have shown that STAT1 can also promote the occurrence of hematological tumors unrelated to IFN.^85^ (5) Regulates the immune system. STAT1 participates in all major histocompatibility complex (MHC)-dependent antigen presentation processes.^86^ In addition, STAT1 is involved in the early development of B cells.^87,88^ The absence of STAT1 or a mutation in the STAT1 gene increases the body’s susceptibility to parasites, bacteria, viruses, etc.^89^

#### STAT2

STAT2 has six complete domains, but STAT2 cannot form homopolymers, nor can it directly bind to DNA.^90^ STAT2 is different from other STATs as the only STAT that does not bind to the original gamma-activated site. STAT2 is activated by type I IFNs (including IFN-α and IFN-β). The biological functions of STAT2 are as follows. (1) Antiviral effects. Interferon stimulation genes are induced to exert the body’s antiviral effect.^91^ (2) Immune regulation.

In STAT2-knockout mice, the lack of type I IFNs autocrine loop and the defective response of T cells and macrophages indicate that STAT2 is essential for regulating the immune response. STAT2 may also be involved in the maintenance of memory cells upon induction by IFN-α.^92^ (3) Regulation of tumorigenesis. STAT2 is highly expressed in ovarian cancer. In addition, STAT2 is associated with the poor overall survival of ovarian cancer and non-small cell lung cancer.^93^

#### STAT3

Similar to STAT1, STAT3 also has two splicing isoforms (STAT3α and STAT3β) with different functions.^94^ STAT3α has a complete domain, while STAT3β lacks 55 amino acids at the C-terminus, replaced by seven amino acid residues.^95,96^ STAT3 is activated when either Y705 or S727 is phosphorylated. Nevertheless, STAT3β lacks S727 and is only activated when Y705 is phosphorylated. STAT3β has better specific DNA-binding activity than STAT3α, but in terms of transcription activity, STAT3α performs better. The transcription factor STAT3 is activated by the IL-6 family members (IL-6, IL-11, IL-31, LIF, CNTF, CT-1, OSM, etc.), IL-10 family members (IL-10, IL-19, IL-20, IL-22, IL-24, and IL-26), and IL-21, IL-27, G-CSF, leptin, and IFN-Is.^55,97–99^ STAT3 has a more complicated role in biological functions. STAT3 is mainly involved in the negative regulation of the immune response, cell growth, differentiation and apoptosis, and tumor occurrence and metastasis. (1) Immune regulation. STAT3 can function in a series of signal transduction processes, such as membrane binding, phosphorylation, and nuclear translocation through a cycle of palmitoylation–depalmitoylation, thereby regulating the differentiation of Th17 cells.^100^ STAT3 also promotes the immunosuppression of tumor-associated macrophages and myeloid-derived suppressor cells.^101,102^ Excessive activation of STAT3 is related to immunosuppression and transformation.^103,104^ (2) Regulation of cell growth, differentiation, and apoptosis. Inhibition of JAK that transduces IL-6 signals to STAT3 may inhibit the expression of the apoptotic protein Bcl-xl, thereby inducing cell apoptosis. Moreover, constitutively activated STAT3 may induce the occurrence of multiple myeloma by inhibiting cell apoptosis.^105^ (3) Regulation of tumorigenesis. The constitutive activation of STAT3 is related to the occurrence of head and neck tumors, breast cancer, non-small-cell lung cancer, colorectal cancer, and hematological tumors. Besides, high expression of STAT3 and IL-6 are closely related to poor chemotherapy sensitivity of triple-negative or high-grade breast cancers.^106^ However, the two splicing isoforms of STAT3 have different functions in the regulation of tumors. STAT3α activation is believed to promote tumor occurrence, while STAT3β inhibits the occurrence of cancer and is considered an effective tumor suppressor.^95,107–109^ (4) Regulation of cancer stem cells (CSCs). The persistent activation of STAT3 maintains the stemness of breast CSCs. There are multiple pathways that promote the survival of CSCs in breast or colon cancer, which include IL-6-JAK1-STAT3, IL-6-JAK2-STAT3, hypoxia-inducible factor-1α-JAK1-STAT3, and retinol-binding protein 4-JAK2-STAT3.^110^

#### STAT4

STAT4 consists of 784 amino acids, and its protein structure is similar to that of other STATs. The cytokines that activate STAT4 mainly include type I IFN, IL-12, and IL-23.^111,112^ In mice and humans, STAT4 plays an essential role in the differentiation and development of Th1 cells and helper follicular T (Tfh) cells. Moreover, STAT4 promotes the germinal center response after a virus attack. Cytokines such as IL-21 produced by Tfh cells are also crucial for the maturation and development of B cells and the isotype conversion of Ig.^113^ Phosphorylation of STAT4 is critical for the humoral immune response.

#### STAT5

The transcriptional activators of STAT5 include STAT5a and STAT5b. The similarity of STAT5a and STAT5b at the amino acid level is 91%. STAT5a is composed of 794 amino acids, and the 694th amino acid is a tyrosine phosphorylation site, while STAT5b is composed of 787 amino acids.^114,115^ STAT5 tyrosine phosphorylation site is the 699th amino acid. Purified STAT5b has a higher DNA-binding capacity than STAT5a.^116^ STAT5a can form tetramers in addition to dimers, while STAT5b binds to DNA in the form of dimers.^117^ The cytokines that activate STAT5 mainly include IL-3, prolactin, and the IL-2 cytokine family (including IL-2, IL-4, IL-7, IL-9, and IL-15). In addition, EGF, EPO, GM-CSF, TPO, GH, and platelet-derived growth factors can also effectively activate STAT5.^54,114,115,118,119^ The biological functions of STAT5 include the following.^120^ (1) Regulation of growth and development. Since it was initially found to nullify the β-casein gene, STAT5a was originally called prolactin-induced mammary gland factor. STAT5-knockout mice present severe defects in mammary gland development and milk secretion, and STAT5b-/- mice exhibit defects in the production of GH.^121^ (2) Regulation of the immune system. STAT5 dimers are essential for survival, STAT5a- and STAT5b-deficient mice exhibit severe defects in lymphatic development and perinatal lethality. However, STAT5a-STAT5b tetramer-deficient mice are viable, whereas had fewer number of T cells, natural killer (NK) cells, and impaired proliferation capacity of CD8+ T cells, and impaired NK cell maturation.^117,122^ (3) Regulation of tumor immunity. After inoculating tumors in immunocompromised mice, the levels of STAT5a and STAT5b of T and B lymphocytes isolated from mice were significantly reduced, indicating that the levels of STAT5 are related to tumor progression.^120,123^ Besides, STAT5 is involved in breast tumorigenesis, and mainly participates in the early development of breast cancer.^106^ (4) Regulation of cell growth, differentiation, and apoptosis. Studies have found that IL-2-induced activation of STAT5 can also lead to an increase in FasL, indicating that STAT5 activation is involved in IL-2-induced activation-induced cell death.^124,125^

#### STAT6

The STAT6 gene encodes 850 amino acids, and the tyrosine phosphorylation site in the STAT6 protein at position 641 marks the activation of STAT6.^126^ however, studies have also pointed out that S407 may be the key phosphorylation site for virus activation of STAT6.^127^ In some cells and tissues, splice variants of STAT6 are evident. STAT6b has a deletion at the amino terminus, and part of the SH2 domain of STAT6c is missing.^128^ STAT6 is mainly involved in the transduction of IL-4 and IL-13 signals.^129^ IL-4 induces activation of STAT6, which is the key to Th2 cells differentiation and immunoglobulin isotypes conversion.^130–132^ Furthermore, IL-4-induced activation of STAT6 in T cells can also inhibit the expression of VAL-4, a member of the family of integrin adhesion molecules, thereby inhibiting the infiltration of CD8+ T cells into tumors.^133^ STAT6 can promote the proliferation and maturation of B cells, mediate the expression of MHC-II and IgE, and play an important role in mast cell activation.^115^ In contrast to other STATs, STAT6 can be activated by viruses without relying on JAK.^127^ STAT6 also induces the expression of homing-related genes in immune cells and plays an important role in innate immunity. For example, the STAT6 dimer induces the expression of CCL2 and recruits T cells, macrophages, and monocytes; CCL26 induces homing of eosinophils/basic granulocytes and NK cells; and CCR6 recruits dendritic cells, B cells, T cells, etc. (Table 2).

**Table 2:** Activated STAT family cytokines and growth factors and STAT-mediated biological functions

STAT	Cytokine and growth factor	Biological functions
STAT1	All interferons, IL-2, IL-6, PDGF, EGF, HGF, TNF, angiotensin II	(1) Regulate cell growth and differentiation; (2) Promote cell apoptosis; (3) Inhibit tumor occurrence; (4) Regulate immune response.
STAT2	Type IIFNs	(1) Type I interferon response mediates the body’s antiviral effect.
STAT3	IL-6 family (IL-6、IL-11、IL-31、LIF 、CNTF 、CT-1 、OSM 、CLCF1) IL-10 family (IL-10、IL-19、IL-20、IL-22、IL-24、IL-26) IL-21、IL-27、G-CSF、Leptin and IFN-Is	(1) Regulates Th17 immune response; (2) Regulates cell growth, differentiation, and apoptosis.; (3) Regulate the occurrence of tumors (promote and inhibit).
STAT4	Type IIFNs, IL-12, IL-23	(1) Regulate the differentiation and development of Th1-type cells and induce Th1-type immune response.
STAT5a, STAT5b	IL-3, Prolactin, IL-2 cytokine family (IL-2, IL-4, IL-7, IL-9 and IL-15) EGF, EPO, GM-CSF, TPO, GH and PDGF IL3, IL-5	(1) Regulate the growth and development of mice; (2) Regulate cell growth, differentiation, and apoptosis; (3) Regulate the production of immune cells (NK cells, T cells, etc.); (4) Related to tumor progression.
STAT6	IL-4, IL-13	(1) Regulate the differentiation of Th2 cells; (2) Regulate the conversion between immunoglobulin isotypes; (3) Promote the proliferation and maturation of B cells, and induce the expression of MHC-II and IgE.

*STAT* signal transducer and activator of transcription, *PDGF* platelet-derived growth factor, *EGF* epidermal growth factor, *HGF* hepatocyte growth factor, *TNF* tumor necrosis factor, *IFN* interferon, *LIF* leukemia inhibitory factor, *CNTF* ciliary neurotrophic factor, *CT-1* cardiotrophin-1, *OSM* oncostatin M, *CLCF1* cardiotrophin-like cytokine-1, *G-CSF* granulocyte colony-stimulating factor, *EGF* epidermal growth factor, *EPO* erythropoietin, *GM-CSF* granulocyte–macrophage colony-stimulating factor, *TPO* thrombopoietin, *GH* growth hormone, *NK* natural killer, *MHC-II* major histocompatibility complex

## Activation and regulation of JAK/STAT signaling pathways

### Canonical JAK/STAT signaling pathway

**Fig. 3:**
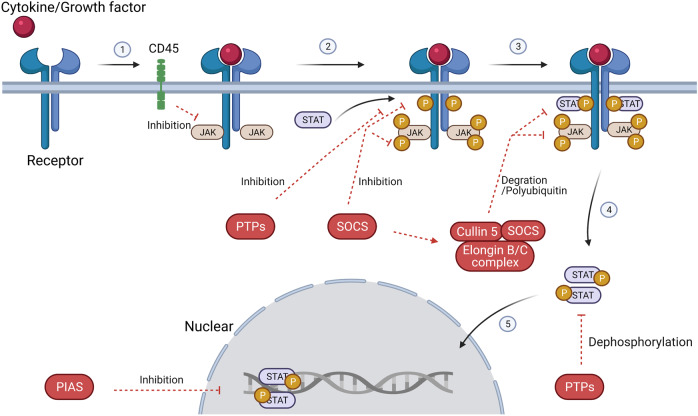
Activation and negative regulation of JAK/STAT signaling pathways. Black arrows indicate the activation process. Red dotted arrows indicated negative regulation. Activation of the JAK/STAT signaling pathway: (1) cytokines and growth factors bind to their corresponding receptors, leading to receptor dimerization and recruitment of related JAKs; (2) JAK activation leads to tyrosine phosphorylation of the receptors and formation of docking sites for STAT; (3) STATs are phosphorylated by tyrosine; (4) STATs dissociate from the receptor to form homodimers or heterodimers; (5) STAT dimers enter the nucleus, bind to DNA, and regulate transcription. Negative regulation of the JAK/STAT signaling pathway: There are three main types of proteins involved in the negative regulation of the JAK/STAT signaling pathway: the PIAS (protein inhibitor of activated STAT), CIS/SOCS (suppressor of cytokine signaling) family, and PTPs (protein tyrosine phosphatase). PIAS mainly interacts with STAT dimers to inhibit STAT binding to DNA, thereby blocking JAK/STAT signal transduction. The CIS/SOCS family negatively regulates the JAK/STAT pathway in three ways: (1) binding to a tyrosine kinase receptor to block the recruitment of STAT; (2) binding directly to JAK to inhibit its kinase activity; (3) forming an elongin B/C-cullin5 complex that degrades JAK or STAT bound to the SOCS protein through polyubiquitination and proteasome degradation. PTPs inhibit the JAK/STAT pathway by interacting with JAK, STAT, or receptors to (1) dephosphorylate the STAT dimer; (2) interact with the receptor to dephosphorylate the related JAK; and (3) in the case of CD45 (a transmembrane PTP) inhibits the phosphorylation of JAK. Created with BioRender.com

The classic JAK/STAT signaling is as follows (Fig. 3): the cell ligand interacts with its receptor to cause receptor dimerization. Nevertheless, gp130,^134^ EpoR,^135,136^ TNF-R1,^137^ IL-17R,^138^, IL-10R,^139^ and GH receptor^140^ etc. can pre-form inactive receptor dimers before binding to the ligands, which may facilitate rapid receptor complex assembly and signal transduction. The connection between the ligand and the receptor induces transphosphorylation of JAK. Activated JAK causes tyrosine phosphorylation of the bound receptor, forming a docking site for STATs. At this docking site, JAK phosphorylates STAT, and then STAT dissociates from the receptor and forms homodimers or heterodimers through SH2-domain–phosphotyrosine interactions. These dimers translocate to target gene promoters, regulation the transcription of the target genes.^4,141^ STAT usually regulates transcription through the following mechanisms: (1) STAT binds to its DNA target site to drive transcription activation. (2) STAT protein may form a transcription complex with non-STAT transcription factors to trigger the transcription mediated by STAT; (3) STAT associates with non-STAT DNA-binding elements to promote STAT-dependent transcription; (4) STAT and non-STAT transcription factors can synergistically activate transcription by binding to clusters of independent DNA-binding sites.

### Noncanonical JAK/STAT signaling pathway

Studies have also shown that JAK/STAT also is involved in nonclassical signal transduction, which is more complicated. Unphosphorylated STAT3 could induce multiple STAT3 target gene expressions without S727 phosphorylation, Lys-685 acetylation and NF-κB contribute to this process. Besides, STATs can be activated not only in the cytoplasm, all STATs except STAT4 can localize to the mitochondrion and promote oxidative phosphorylation and membrane permeability, STAT3 can localize to the endoplasmic reticulum and help to resist apoptosis induced by oxidative stress.^142^ A portion of the unphosphorylated STAT pool is located on heterochromatin related to the allelic autosomal gene heterochromatin protein-1 (HP1) in the nucleus. JAK or other kinases induce the activation of STAT and cause HP1 to dissociate from heterochromatin, and then, phosphorylated STAT binds to cognitive sites on autosomes to regulate gene transcription. This atypical JAK/STAT signal transduction is essential for maintaining the stability of heterochromatin.^143–145^ In a study of Drosophila, it was found that the phosphorylation of STAT can cause HP1 to break away from heterochromatin, thereby destroying the stability of heterochromatin, and the instability of heterochromatin may promote the occurrence of tumors.^145–148^ Some researchers have discovered that atypical JAK/STAT signal transduction patterns exist in mammals. For example, MHC high-order chromosomal remodeling is caused by IFN-induced STAT1 activation;^149^ other studies have shown that activation of JAK3-STAT5 causes chromatin remodeling at the Ifng locus during Th1 cell differentiation.^150^ Interestingly, JAK protein can also be activated by tumorigenic tyrosine kinases independent of cytokine receptors.^151^ For example, the proto-oncogene v-Abl of Abelson murine leukemia virus constitutively activates the JAK/STAT pathway by regulating the interaction between suppressors of cytokine signaling (SOCS)-1 and JAK in the JAK/STAT pathway.^152^ The oncogenic fusion chimeric protein nucleophosmin-anaplastic lymphoma kinase induces the inactivation of SH2-containing protein tyrosine phosphatase-1 (SHP-1) to inhibit the degradation of JAK3 and enhance the signal transduction initiated by JAK3-STAT3, which may lead to the occurrence of anaplastic large cell lymphoma.^153^ The BCR-ABL fusion gene exerts anti-apoptotic effects. BCR-ABL can synergize with hematopoietic growth factors through low constitutive phosphorylation of JAK protein, thereby regulating STAT activation.^154^ Similarly, STAT can also be activated by other non-receptor tyrosine kinases or directly activated by receptors independent of JAK. c-Src tyrosine kinase can constitutively activate STAT3, which increases the possibility of the STAT signaling pathway regulating tumor-related gene expression.^155^ Epidermal growth factor receptor can directly activate STAT1, STAT3, and STAT5, furthermore, STAT5 can be directly activated by the platelet-derived growth factor receptor.^99,156,157^

### Positive regulation of JAK/STAT signaling

In addition to the main components of the JAK/STAT signaling pathway, many related proteins play indispensable roles in STAT-dependent transcription and JAK–STAT interactions with other signaling pathways. Upon co-stimulation of glucocorticoids and prolactin, activated STAT5 and glucocorticoid receptor (GR) form a complex. GR acts as a transcriptional coactivator of STAT5 to promote STAT5-dependent transcription.^158^ Moreover, CBP and p300 act as auxiliary activators of STAT1α to regulate the response of JAK/STAT, but this regulation can be realized by integration of common transcripts of the JAK/STAT and other signaling pathways.^159^ Another cytoplasmic protein, Nmi, may promote the activation of STAT1 and STAT5 through the recruitment of STAT1 and STAT5 by CBP. In vitro GST pull-down assay results showed that STATs except STAT2 could interact with Nmi.^66^ Some adaptor proteins can also promote the JAK/STAT signaling pathway. The SH2 protein subfamily composed of lymphocyte adaptor protein (Lnk), SH2-B, and APS has potential adaptor functions. SH2-2B can promote the activation of JAK2 induced by GH, while APS is a negative regulator of the JAK/STAT signaling pathway.^160^ signal transducing adapter molecule is a transduction adapter molecule containing an SH3 domain and one ITAM domain. It can interact with JAK2 and JAK3 through its ITAM domain to enhance IL-2 and GM-CSF-mediated C-myc transcription.^161^

### Negative regulation of JAK/STAT signaling

Many negative regulators are involved in the regulation of JAK/STAT signal transduction. They maintain the balance and steady state of the JAK/STAT pathway. There are three main types of negative regulation of the JAK/STAT signaling pathway: protein inhibitor of activated STAT (PIAS), SOCS/CIS family members, and PTPs (protein tyrosine phosphatases) (Fig. 3).

#### SOCS/CIS family

The SOCS protein family are intracellular proteins that include CIS, SOCS1, SOCS2, SOCS3, SOCS4, SOCS5, SOCS6, and SOCS7. Activated STATs dimerize and enter the nucleus to induce SOCS expression and the SOCS proteins bind to phosphorylated JAK and its receptor to negatively regulate the JAK–STAT signaling pathway. SOCS mainly negatively regulates the JAK/STAT pathway in three ways. (1) It binds to the phosphotyrosine on the receptor to prevent STAT recruitment to the receptor; CIS binds stably to the tyrosine-phosphorylated β chain of the IL-3 receptor and the tyrosine-phosphorylated EPO receptor.^162^ (2) It directly and specifically binds to JAK or its receptor to inhibit the kinase activity of JAK. For example, SOCS3 binds to JAK and its receptor gp130 simultaneously. SOCS3 targets the IL-6 family cytokine-receptor complex containing the signaling receptor gp130. After the SH2 domain of SOCS3 binds to phosphorylated Tyr759 of gp130, SOCS3 Ig-like receptors (KIR) bind to gp130-related JAK in a nonphosphorylation-dependent manner. Upon SOCS3 binding, the substrate-binding groove of JAK is hidden, inhibiting the JAK/STAT signaling pathway.^163^ The SH2 domain of the SOCS1 protein can target the activation loop of JAKs, and SOCS1 can also directly inhibit JAK tyrosine kinase activity through KIR.^164^ (3) The SOCS proteins interact with the elongation protein B/C complex through its C-terminal SOCS box and simultaneously binds to the cullin5 scaffold protein to form an elongation protein-cullin-SOCS3 E3 ubiquitin-linked enzyme complex.^165^ This complex undergoes polyubiquitination, and the proteasome degrades signaling factors such as JAKs and STATs that bind to SOCS, thereby blocking signal transduction. Cullin5 also contains a Really Interesting New Gene (RING) domain that can bind to the protein Rbx2, which can interact with the E2 ubiquitin-conjugating enzyme. SOCS not only plays a simple negative feedback function but also plays an important role in the immune response and inflammation regulation. Experimental data in SOCS1 knockout mice showed that SOCS1 inhibits Treg cells secretion of IFN-γ by regulating STAT1, and the inhibition of IFN signaling prevented atherosclerosis.^166^ CIS does not interact with JAKs and therefore does not inhibit JAKs. It was initially identified as a negative feedback regulator of STAT5. Moreover, overexpression of SOCS3 in Ba/F3 cells can inhibit the activation of STAT5. SOCS3 inhibits Th1 cells and promotes Th2 production by inhibiting IL-12-mediated STAT4 activation. The loss of SOCS3 can also inhibit the production of Th1 cells and Treg cells by promoting the production of IL-10 and transforming growth factor β (TGFβ).^166^

#### PIAS

PIAS is a family of transcriptional regulators. There are four PIAS family members that are found in mammals, namely, PIAS1 (also known as PIASx, and two splice variants PIASx-α and PIASx-β), PIAS2, PIAS3 (with splice variant PIAS3b), and PIAS4 (also known as PIASy with splice variant PIASyE6). The PIAS homolog dPIAS/Zimp was identified in Drosophila,^167^ and PIAS-related proteins SIZ1 and SIZ2 also exist in yeast.^168^ PIAS was initially found to be an inhibitor of STAT, and PIAS1 and PIAS4 can interact with STAT1, PIAS3 and PIAS1 interact with STAT3 and STAT4, respectively. PIAS only interacts with STAT dimers formed after being phosphorylated by JAK, and does not interact with STAT monomers. PIAS mainly regulates transduction through the following mechanisms. (1) Blocking the DNA-binding activity of transcription factors. For example, PIAS1 and PIAS3 block JAK/STAT signal transduction by blocking STAT and DNA-binding activity.^37,169^ (2) Promoting transcription factor sumoylation. Research results show that PIAS1 can interact with Lys703 on STAT1.^170^ (3) Recruiting other co-regulatory factors, namely, PIAS1 and PIAS4, through the recruitment of the co-inhibitory molecule histone deacetylase, which prevents STAT binding to DNA and leads to transcription-activation failure.^171^ (4) Chelating transcription factors to form the subnuclear structures of repressor complexes to regulate transcription.^172^ PIAS also acts as a SUMO (small ubiquitin-related modifier) E3 ligase, which can regulate many cellular processes through protein ubiquitination; however, there is still debate on whether the SUMO E3 ligase activity of PIAS regulates STAT signaling. PIASx-α can act as an E3 ligase to modify the Lys703 SUMO of STAT1. However, interestingly, mutating Lys703 to Arg can eliminate the SUMO modification, but the activation of STAT1 and PIAS1 inhibition of STAT1 signaling is not affected.^170^ In contrast to these findings, Ungureanu et al. revealed that the same mutation caused an increase in IFN-γ-mediated transactivation of STAT1, leading to increased activation of STAT1.^173^ A large number of genetic studies have also verified the physiological role of PIAS in the gene regulation mediated by the JAK/STAT signaling pathway. JAK/STAT transduction activity is elevated when PIAS was knocked out, which leads to the formation of hematological tumors, and PIAS1 selectively regulates IFN-β and IFN-γ inducible genes by interfering with the recruitment of STAT1 to gene promotors.^174^ However, how the SUMO E3 ligase activity of PIAS regulates STAT activity in vivo and the physiological role of STAT-mediated gene regulation need further research and elucidation.

#### PTPs

The JAK/STAT signaling pathway can also be negatively regulated by PTPs. The SH domain in PTPs can bind to signaling molecules, activated receptors, and JAK to dephosphorylate a substrate. PTPs can dephosphorylate STAT and inhibit its activity, and inhibit JAK/STAT signal transduction. For example, the nuclear isoform TC45 of T-cell PTPs has been extracted from HeLa cells. Nuclear TC45 dephosphorylates and inactivates STAT dimers in the nucleus.^175^ SH2-containing protein tyrosine SHP-1 is also an important member of the PTP family. When it is activated by GH and transfers to the nucleus, SHP-1 can dephosphorylate STAT5b.^176^ PTPs not only act on activated STAT but can also dephosphorylate JAK and block the JAK/STAT signaling pathway. The transmembrane PTP CD45 can inhibit IL-3-induced JAK2 phosphorylation and negatively regulate JAK/STAT signal transduction, thereby inhibiting IL-3-mediated cell proliferation.^177^ PTP1B can act on specific sequences in the JAK activation loop in the cytoplasm, dephosphorylating JAK2 and TYK2, but it has also been reported that the main target of PTP1B in the suppression of JAK/STAT signaling is STAT5.^178^ Other PTPs can also act on ligand-receptor complexes. For example, hematopoietic protein tyrosine phosphatase SH-PTP1 can bind to pY429 in the cytoplasmic region of the EPO receptor, thereby mediating dephosphorylation and inactivation of JAK2. After adding IFN-α, SHP-1 can also reversibly bind to IFN-α receptors and selectively regulate JAK/STAT signal transduction in mice.^179^ SH2-containing protein tyrosine phosphatase-2 (SHP-2) can negatively regulate the cytotoxic effect of IFN on the overactivation of STAT to promote cell growth, but the specific role of SHP-2 is related to a part of the JAK/STAT signaling pathway that remains to be studied.^180^

### Signaling cross-talk between JAK/STAT and other pathways

**Fig. 4:**
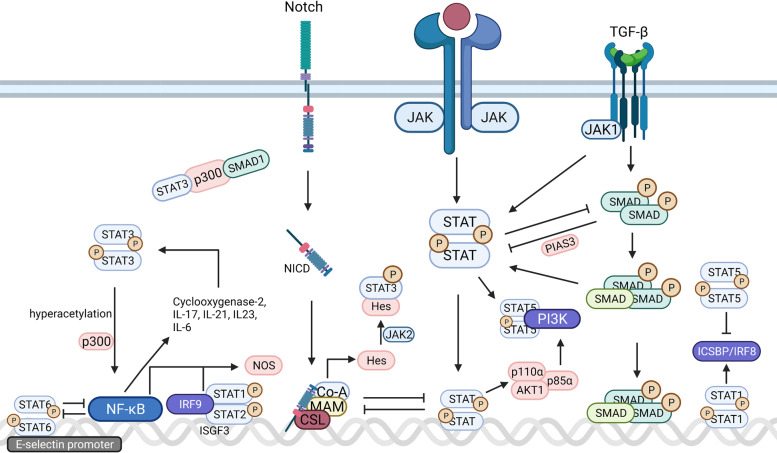
Signaling cross-talk between JAK/STAT and other pathways. (1) STAT3-p300- SMAD1 forms a complex to induce astrocyte differentiation; (2) JAK1 binds to TGFβRI and activates STAT3; (3) TGF-β also activates STAT3 via SMAD-dependent manner. (4) STAT3 inhibits SMAD3–SMAD4 complex formation and suppress SMAD3-DNA binding; (5) SMAD3 inhibits STAT3 activation via recruiting PIAS3 to STAT3; (6) TGF-β blocks IL-12 mediated JAK2 and TYK2 tyrosine phosphorylation; (7) Notch signaling suppresses JAK/STAT activation by interfering with STAT translocation to the DNA domain, and signals of JAK/STAT inhibited Notch signaling conversely. (8) Hes proteins directly bind to STAT3 and induce phosphorylation by recruiting JAK2; (9) STAT5 direct interacts with PI3K; (10) STAT5 upregulates the expression of *p85*α (*Pik3r1*), *p110α (Pik3ca)*, and *AKT1*; (11) STAT3 drives the hyperacetylation of RelA via interacting with p300; (12) cyclooxygenase-2, IL-17, IL-21, and IL-23 encoded by NF-κB activate STAT3; (13) NF-κB preceded ISGF3 (a complex containing STAT1, STAT2, and IRF9 subunits) at the Nos2 promoter, thus regulating nitric oxide synthase expression; (14) IRF9-STAT1-STAT2 trimeric complex induce gene expression; (15) STAT1 regulates IRF8 synthesis; (16) STAT5 suppresses IRF8 activity; (17) STAT1 and IRF1 synergistic induce IFNγ-induced gene transcription; (18) IRF8 increases IFNγ-induced gene transcription mediated by STAT1 and IRF1. Created with BioRender.com

Cross-talk between components in the JAK/STAT pathway and those in other pathways is complex, occurs at various levels, and involves diverse molecules, such as a receptor, JAK, STAT, and gene transcription factors (Fig. 4). These cross-talk activities play vital roles in pluripotency and differentiation transcription program, immune regulation, and tumorigenesis.

#### The TGFβ signaling pathway

The TGFβ family consists of TGFβs, bone morphogenic proteins (BMPs), activins, and Nodal. The TGFβ signaling pathway regulates a wide range of biological activities in various cell types, such as embryonic development and cell homeostasis. SMAD proteins are pivotal intracellular effectors or modulators of the TGFβ family. SMADs and STATs are often combined in the same transcription complex. For example, LIF-STAT3 and BMP2-SMAD1 synergistically induce primary fetal neural progenitor cells to differentiate into astrocytes. STAT3 interacts with p300 at its amino terminus, SMAD1 interacts with p300 at its carboxyl terminus. STAT3 and SMAD1 form a complex linked by p300, which contributes to the astrocyte differentiation.^181^ It has been reported that the TGFβ signaling pathway regulates the JAK/STAT pathway in both a positive and negative manner, depending on the cell type and protein status.^182^ In pancreatic ductal carcinoma, tumor-secreted TGFβ antagonizes the upregulation of LIF induced by IL-1 by downregulating the expression of IL-1R1 and promoting the differentiation of cancer-associated fibroblasts into myofibroblasts, thereby inhibiting JAK/STAT signaling.^183^ In contrast, in hepatocytes, hematopoietic stem cells (HSCs), and hepatoma cells, TGFβ can potentiate IL-6 mediated STAT3 activation. In liver fibrosis, JAK1 is a constitutive TGFβRI-binding protein; thus, STAT3 is activated directly through JAK1 within minutes of TGFβ stimulation in a SMAD-independent manner. TGFβ also provokes a second phase activation of STAT3, which depends on SMADs, de novo protein synthesis, and JAK1. Activated SMAD and STAT3 bind to their respective DNA domains in the *JUNB* promoter to enhance the expression of TGFβ related genes.^184^ In addition to the cooperative function between SMAD3 and STAT3, it is reported that STAT3 can also attenuate SMAD3–SMAD4 complex formation and suppress SMAD3-DNA binding. Furthermore, SMAD3 can recruit PIAS3 to STAT3, thus inhibiting STAT3 activation.^185^ The phosphorylation status of SMAD3 and STAT3 determines whether the relationship between them is cooperative or antagonistic.^184^ In T lymphocytes, TGFβ blocked IL-12-mediated JAK2 and TYK2 tyrosine phosphorylation and STAT3 and STAT4 activation in T lymphocytes, resulting in decreased T-cell proliferation and diminished IFN-γ production.^186^

#### The MAPK signaling pathway

Mitogen-activated protein kinase (MAPK) cascades are complex signaling pathways that regulate various cellular activities, including inflammation, apoptosis, proliferation, and differentiation. There are three major subfamilies in the MAPK family: extracellular-signal-regulated kinases (ERK), c-jun N-terminal kinase or stress-activated protein kinases (JNK or SAPK), and MAPK14. As a well-known pathway participating in cellular activities, the JNK signaling pathway is involved in both apoptosis and cancer cell survival. For example, JNK mediates the compensatory cell proliferation in tumors. Nevertheless, via regulating the *fos* gene and pro-apoptotic *hid* gene, the JAK/STAT pathway and downstream effector *Zfh2* promotes JNK-mediated cell survival, and inhibits JNK-mediated cell apoptosis.^187^

#### The Notch signaling pathway

The Notch signaling pathway is evolutionarily conserved and extensively controls cell processes, such as proliferation, differentiation, and cell death. The crosstalk between components in the Notch signaling pathway and those in the JAK/STAT signaling pathway is mainly studied in organ development. In Drosophila posterior foregut development, analysis of gene expression mediated by a model Notch response element revealed that the JAK/STAT signaling pathway is required for the expression of Notch-dependent genes in the foregut, which indicated the function of the JAK/STAT pathway controlling cell movement during embryonic foregut development, the molecular mechanism remains to be determined.^188^ In Drosophila intestinal stem cells (ISCs), researchers found that the JAK/STAT pathway regulated ISCs proliferation, and that this effect may have been inhibited by Notch signaling at the transcriptional level. This type of antagonistic relationship ensures a balance between proliferation and differentiation of ISCs.^189^ In the development of drosophila follicle cell patterning, Notch signaling suppresses JAK/STAT activation by interfering with STAT translocation to the DNA domain, and signals of JAK/STAT inhibited Notch signaling conversely. Thus, the continuous balance of two pathways specifies the identity of different types of follicle cells.^190^ In the development of the central nervous system, Hes proteins, downstream effectors of Notch, directly bind to STAT3 and induce phosphorylation by recruiting JAK2, indicating that Hes proteins may be non-receptor scaffold proteins that facilitate JAK2 phosphorylation of STAT3.^191^ In breast cancer, the Notch signaling pathway is often hyperactivated, noncanonical Notch signaling upregulates IL-6 expression, then activates downstream JAK/STAT, and Notch-mediated IL-6 upregulation occurs only when p53 was mutated or lost. In addition, activation of IL-6 by Notch required the IKKα/IKKβ function (inhibitor of NF-κB kinase subunit alpha and beta, respectively). IKKα and IKKβ are two proteins in the NF-κB signaling pathway.^192^

#### The PI3K/AKT/mTOR pathway

The phosphatidylinositol 3-kinase (PI3K)/AKT/mammalian target of rapamycin (mTOR) pathway plays a vital role in most cellular processes, such as proliferation, adhesion, migration, and invasion. In human melanoma cells, PI3K negatively regulates STAT activity.^193^ In mammary epithelial cells, the JAK2/STAT5 pathway controls mammary epithelial cell survival and death through direct interaction with the p58α regulatory subunit of PI3K and upregulation of the expression of *p85*α (*Pik3r1*), *p110α (Pik3ca)*, and *AKT1.*^194^ In cytokine-receptor-like factor 2-rearranged B-precursor acute lymphoblastic leukemia, increased pJAK2, pSTAT5, and pS6 levels were observed in patient samples. JAK inhibitors inhibited both the JAK/STAT and PI3K/mTOR pathways, which suggests an interconnection between them. Nevertheless, for full elucidation of the mechanism, additional work is needed.^195^

#### The NF-κB signaling pathway

The NF-κB family comprises five members: p50, p52, p65, c-RelA, and RelB. NF-κB dimers bind to DNA sites called κB sites to modulate gene expression. NF-κB regulates a large variety of cellular responses, especially throughout the immune system.^196^ The cross-talk between the JAK/STAT signaling pathway components and the NF-κB signaling pathway components is extensive. NF-κB can induce the expression of a variety of inflammatory mediators and is a core transcription factor in various immune responses. Therefore, it is believed that NF-κB can induce malignancy and antitumor immunity through simultaneous inflammation.^197^ Some factors regulated by STAT3 also play essential roles in the tumor microenvironment.^105,198–200^ When it was found that NF-κB and STAT3 in tumor cells were activated simultaneously, people connected the two.^201^ Among these factors, IL-6 is an important factor that links the NF-κB signaling pathway with STAT3. As we mentioned above, IL-6 and its ligand can effectively activate STAT3; and the target gene of NF-κB encodes IL-6. STAT3 plays a vital role in the activation of the NF-κB pathway. In cancer cells and tumor-related haematopoietic cells, constitutively activated STAT3 drives the hyperacetylation of RelA, mediated by interactions with p300, thereby prolonging NF-κB nuclear retention and promoting the activation of NF-κB.^202^ In addition, cyclooxygenase-2, IL-17, IL-21, and IL-23 encoded by NF-κB can activate STAT3 in various ways.^200,203,204^ IL-4-mediated STAT6 activation plays a crucial role in inflammatory gene inhibition, partly because STAT6 acts as an antagonist of NF-κB upon the binding of the E-selectin gene promoter.^205^ Moreover, NF-κB preceded ISGF3 (a complex containing STAT1, STAT2, and IRF9 subunits) at the Nos2 promoter, thus regulating nitric oxide synthase expression.^206^

#### The IRF family

The IRF family includes nine members: IRF1, IRF2, IRF3, IRF4/ICSAT/PIP/LSIRF, IRF5, IRF6, IRF7, IRF8/ICSBP, and IRF9/ISGFγ. These factors were initially identified as transcriptional regulators of type 1 interferon. Further studies revealed more functions of the IRF family in addition to their functions in the IFN system, such as immune cell development, innate immune responses, and tumor suppression.^207^ Cross-talk between IRFs and STATs includes both direct physical binding and indirect gene regulation. For example, IRF9 physically binds to STAT1-STAT2 heterodimer, and this trimeric complex binds to a composite DNA element comprising binding sites for both STAT1 and IRF9.^208^ STAT1 stimulates the transcription of IFNγ-inducible genes, and IFN consensus sequence binding protein (ICSBP/IRF8) is an IFNγ-inducible gene. Thus, STAT1 regulates IRF8 synthesis.^209^ Conversely, IRF8 increases IFNγ-induced gene transcription mediated by STAT1 and IRF1.^210^ IRF can be negatively regulated by STAT. For instance, STAT5 suppresses IRF8 during the plasmacytoid dendritic cell development.^211^

## The JAK/STAT pathway in human diseases

The JAK/STAT pathway is a highly conserved pathway of signal transduction. It regulates multiple cellular mechanisms associated with varieties of diseases development. Dysregulation of the JAK/STAT pathway is associated with various diseases. For example, JAK2^V617F^ mutation frequently occurs in myeloproliferative neoplasms (MPN). More frequently, the JAK/STAT pathway serves as a mediator of abnormally elevated cytokines to induce gene transcription. Furthermore, inhibitors of JAK/STAT have been effective in treating multiple diseases, such as rheumatoid arthritis (RA) and systemic lupus erythematosus (SLE), which shows that JAK/STAT is important in disease development (Table 3).^212–214^

**Table 3:** Mutation or overexpression of JAK/STAT at different diseases

Gene	Mutation	Overexpression	Disease
JAK1	JAK1	——	Primary mediastinal B-cell lymphoma
	JAK1	——	Hepatocellular carcinoma
	——	JAK1	Hair loss
	——	JAK1	Atopic dermatitis
	——	JAK1	Age-related frailty
	——	JAK1	Colorectal cancer
JAK2	JAK2 (JAK2 V617F)		Myeloproliferative neoplasms
	——	JAK2	Hodgkin lymphoma
	——	JAK2	Rheumatoid arthritis
	——	JAK2	Atopic dermatitis
	JAK2 (V615L and M532V)	——	Lung tumor
JAK3	JAK3 (L156P, E183G, R172Q)	——	T-cell leukemia/lymphoma
	JAK3	——	Natural killer T-cell lymphoma
	JAK3 (A572V and A573)	——	Severe combined immunodeficiency syndromes
	JAK3 (A1090S)	——	Lung tumor
STAT3	STAT3	——	Job’s syndrome
	——	STAT3	Rheumatoid arthritis
	——	STAT3	Cervical Cancer
	——	STAT3	Bladder cancer
STAT6	STAT6		Primary mediastinal B-cell lymphoma

*JAK* Janus kinase, *STAT* signal transducer and activator of transcription

### Malignancies

#### Hematological malignancies

Abnormal amplification and recruitment of blood cells lead to hematologic malignancies. The normal actions of the JAK/STAT pathway rely on various components. Thus, basic molecular alterations, such as those caused by gain-of-function mutations in different components (JAK, STAT) and extensive expression (cytokine receptors, JAK), may result in aberrant activation of a signaling cascade. JAK2 acts as an essential mediator in HSCs by transmitting signals from TPO and activating downstream stem cell factors.^215,216^ JAK2 mediates myelopoietic formation at different stages through its interactions with various receptors (e. g. EPO, TPO, and GM-CSF).^135^ In addition, the combined actions of JAK1 and JAK2 are crucial for lymphopoiesis. Both JAK1 and JAK3 can bind to IL-2R, IL-4R, IL-7R, and IL-15R.^34,217^ Gain-of-function mutations in four Janus kinases play roles in hematologic malignancies. The majority of these alternations appear to be point mutations of varying frequency in different JAK members. JAK1 mutations are the most frequent in T-cell acute lymphoblastic leukemia (6.5–27%), followed by B-cell acute lymphoblastic leukemia (1.5%),^218–220^ indicating that JAK inhibitors are necessary to treat hematological disease.

##### Hodgkin lymphoma

Classical Hodgkin lymphoma (cHL), primarily derived from germinal central B cells, represents a case of successful treatment.^221^ Eighty percent of patients with Hodgkin lymphoma achieve complete remission by using recently combined modality therapies. Despite high cure rates in adolescents and young adults, treatment-related toxicity and long-term morbidity remain a significant challenge in the clinic.^221^

Previous studies revealed that cHL patients experience a recurrence in some genomic lesions, associated with persistent activation of the NF-kB and JAK–STAT signaling pathways with proinflammatory and anti-apoptotic features.^222^ Gain-of-function mutation of STAT6 is evident in most patients with cHL (~80%).^223,224^ Furthermore, when STAT6 is mutated, the mutant maintains tumor cell survival and growth in conjunction with unidentified SOCS1 variants by inducing an anti-apoptotic response.^225^ JAK2/STAT6 signaling is activated by lymphotoxin-a produced by cHL cell lines, inducing target gene expression to promote the immunosuppressant microenvironment and lineage ambiguity in cHL.^225^ cHL cells exhibit an aberrant cytokine level that is essential for the proliferation of Hodgkin and Reed/Sternberg cells and a favorable environment for tumor cells. Constitutive activation of the JAK/STAT pathway may be associated with increased cytokine and receptor expression in cHL. Moreover, the role of the JAK/STAT pathway in immune evasion by mediating PD-L1/L2 expression has been reported in Hodgkin lymphoma. Chromosome 9p24.1/PD-L1/PD-L2 mutation upregulates PD-1 ligands and PD-L1 on the membrane through JAK/STAT signaling.^226–228^

##### Natural killer/T-cell lymphoma

Current knowledge on natural killer/T-cell lymphoma (NKTCL) is insufficient to understand its molecular mechanisms well. Furthermore, few therapeutic approaches are available to patients with NKTCL. To date, simple dependence on multiagent chemotherapy and localized radiotherapy has shown poor benefits. With technical progress, more disease-related genes have been found in NKTCLs. The role of the JAK/STAT pathway in promoting the maturation of HSCs has been gradually acknowledged. Increasing evidence shows that a persistently active JAK/STAT pathway may be caused by mutations in JAK gene domains, and they probably lead to the pathogenesis of lymphocyte-related malignancies, including T-cell acute lymphoblastic lymphoma/leukemia, cutaneous TCL, mantle cell lymphoma, and acute megakaryoblastic leukemia.^218,229–234^ JAK3 mutation has been reported in many other cancers, such as breast, stomach, and lung cancer.^219,235^ Concordant with these results, the samples from patients with NKTCL tumor were found to express JAK3 mutations.^236^ In addition, Cornejo and colleagues showed that transplanting JAK3-mutant bone marrow cells into C57BL/6 mice induced continuous activation of the JAK/STAT signaling pathway, resulting in the generation of aggressive T-cell lymphoproliferative disorders. These data suggest that JAK3-activating mutations may be involved in the development of NKTCLs.^237^

##### Myeloproliferative neoplasm

Myeloproliferative neoplasm (MPN) refers to a group of disorders whose distinctive feature is an extensive expansion of one or more blood cell types, such as white blood cells, red blood cells, and platelets. Patients with MPN may experience thrombohemorrhagic complications. MPN may develop into myelofibrosis (MF) or acute myeloid leukemia (AML), resulting in severe symptoms and a reduced life span. JAK2^V617F^ is the most frequent genetic alteration, whose expression is different in PV (>95%) and ET/PMF (50-60%).^238–241^ In cells carrying JAK2^V617F^, a high-frequency mutation, the inhibitory functions of the JH2 pseudokinase domain are disrupted, resulting in overactivation of the JAK/STAT pathway.^242^ JAK2^V617F^ in megakaryocytes plays a vital role in maintaining the myeloproliferative state of both mutant and non-mutant hematopoietic cells. Excessive proliferation of cells can lead to increased erythropoiesis and fibrosis. The lack of megakaryocytes in JAK2^V617F^ and MPL^W515L^ BMT models leads to significantly alleviated polycythemia and leukocytosis,^242^ indicating that the activation of the JAK/STAT pathway in megakaryocytes is positively linked with myeloproliferation and promotes MPN progression. Aging patients may acquire more frequent mutations of JAK. It is hypothesized that increasing age can be a crucial risk factor for MPN progression.

A majority of patients with MPN present chronic inflammation with enhanced circulating proinflammatory cytokines. It is well-known that continued inflammation may contribute to the progression of MPN.^239^ Thus, the activity of the JAK/STAT pathway may be elevated in response to increases in the levels of proinflammatory cytokines.^243^ Previous studies showed that activated STAT3 proteins could promote cytokine production in a variety of cancers.^244^ Using a JAK2 inhibitor to treat mice with MPN resulted in reduced cytokine levels and attenuated systemic symptoms.^245^ In MPNs, abnormal activation in JAK/STAT signaling is commonly accompanied by mutations in tyrosine kinases. It is well-known that TPO stimulation activates JAK2-STAT3/5.^246^ With further investigation about MPN, the importance of the Lnk has been gradually realized in the field. Lnk as a member of adaptor protein has a negative effect on signaling pathways activated by TPO-R/MPL in either megakaryopoiesis or HSCs.^247–250^ The lack of Lnk leads to significant interference in the hematopoietic function of mice, including a threefold increase in white blood cells and platelets in the circulation, the accumulation of B cells with different states in the bone marrow and spleen, and the expansion of HSCs.^247,248,251^ Data from biochemical experiments implicate that in response to TPO stimulation, the SH2 domain of Lnk interacts with the phosphorylated tyrosine residue 813 (Y813) of JAK2, which makes JAK2 activation suppressed to constrain the quiescence and self-renewal of HSCs. In addition, the published studies reveal that the deficiency in Lnk has shown advanced JAK/STAT signaling in a cytokine-independent manner and the increased ability of oncogenic JAK2 to promote the expansion of myeloid progenitors both in vitro and in vivo.^252^ Moreover, JAK inhibitors inhibit Lnk-deficient cell lines, suggesting that the treatment of JAK2 inhibitors may be a novel choice for MPN patients with Lnk deficiency.

#### Hepatocellular carcinoma

Hepatocellular carcinoma (HCC) ranks third as the most common cause of cancer-related death worldwide.^253,254^ Various factors contribute to the pathogenesis of this cancer, including viral infection, particularly hepatitis B virus (HBV), continuous alcohol consumption, and aflatoxin B1 contaminated food.^255,256^ Despite acquiring the remarkable improvement in understanding risk factors of HCC, the current theories explaining the molecular mechanism of HCC have not been verified.^257^ Thus, deeper exploration needs to be conducted to find better treatments.

SOCS3 can block various cytokine signaling pathways, including the JAK/STAT, and NFκB signaling pathways,^258,259^ and sustain normal immune reactions. Hypermethylation of SOCS3 has been found in multiple malignant diseases, such as lung cancer, head and neck cancer, and prostate cancer.^260–262^ Niwa et al. reported that 33.3% of HCC tissues exhibited hypermethylated SOCS3.^263^ Furthermore, long noncoding RNA promotes HCC progression,^264^ most likely via activation of the JAK/STAT pathway. LINC00346, an intergenic lncRNA located on chromosome 13q34, is upregulated in HCC and promotes tumor cell growth by decreasing the cell apoptosis rate and increasing the cell proliferation rate, which depends on the level of LINC00346 and the activated JAK/STAT signaling pathway.^265^ The phosphorylated transcription factor STAT3 significantly contributes to cancer growth and recurrence. Sorafenib, a Food and Drug Administration (FDA)-approved first-line drug for advanced HCC treatment, induces HCC cell death. STAT prevents the anti-apoptosis effect of sorafenib by modulating Mcl-1 expression.^266–268^ Moreover, STAT3 partially contributes to the sensitivity of HCC cells to sorafenib-mediated cell death.^269^ In contrast, some cytokines that do not activate cytokine receptors negatively regulate HCC progression by inhibiting the JAK/STAT pathway. For example, angiopoietin-like protein (ANGPTL1) acts as a tumor suppressor, not only inhibiting STAT3/Bcl-2–driven anti-apoptotic signals to promote apoptosis but also downregulating certain transcription factors (e.g., SNAIL and SLUG) to suppress cell migration and invasion.^270–272^ Many studies have revealed that DNA hypermethylation in promotors can be associated with tumor suppressor gene dysfunction, leading to HCC development and progression.^273,274^ Thus, the downregulation of ANGPTL1 is insufficient to inhibit STAT3 signaling.^275^

### Inflammatory and immune diseases

#### Systemic lupus erythematosus

SLE, caused by an aberrant autoimmune response,^276^ is a complex immune disorder that can result in inflammation of multiple tissues or organs in the body and in most cases affects the kidneys. A common characteristic of SLE is recognition of specific autoantigens and against which it produces multiple autoantibodies.^277^ Lupus nephritis is a poor prognostic indicator for patients with SLE.

Cytokines play a central role in the pathogenesis of SLE.^278,279^ A wide range of cytokines are considered immunopathological in the initiation and development of human SLE, such as IFNs, TNF, IL-6, IL-10, and IL-12.^280,281^ Increased serum IFN and expression of IFN-inducible genes mediated by the JAK/STAT pathway is thought to be pivotal in the molecular pathogenesis of SLE. JAK1 and TYK2 are downstream signals of IFN. Moreover, TYK2 polymorphism is closely linked with SLE.^282^ CXCR4, a vital chemokine receptor with multiple immune functions, is significantly upregulated in patients with SLE.^283,284^ CXCR4 endocytosis is mediated through IL-21 and B-cell receptor interactions, which are likely dependent on the JAK/STAT signaling pathway.^285^ Furthermore, CXCR4 undergoes tyrosine phosphorylation by JAK2 and JAK3.^286^ These data indicate that the activated JAK/STAT pathway is tightly associated with an abnormal increase in CXCR4 in patients with SLE. In addition, increased infiltration of immune cells in renal interstitium and glomeruli contributes to disease progression and development through overexpression of the JAK/STAT pathway. It is reported that JAK inhibitors significantly suppress the infiltration and cytokine production of T cells.^287,288^

#### Atopic dermatitis

Atopic dermatitis is a chronic inflammatory skin disease caused by aberrant autoimmune responses. The prevalence ranges from 5 to 20%.^289,290^ The incidence tends to be higher in children than adults. Th2 differentiation, important for the initiation and development of atopic dermatitis, may be regulated by activating the JAK/STAT pathway. Thus, the JAK/STAT pathway is linked to inflammation and pruritis in atopic dermatitis. Many therapies have been applied to improve patients’ quality of life, including phototherapy, systemic corticosteroids, systemic immunosuppressants, and monoclonal antibody dupilumab.^291^ Their insufficient effect and potential risks still need to be addressed.^291^ An increase in cytokines, such as Th2, Th22, Th1, and Th17 secreted cytokines, has been identified in atopic dermatitis skin lesions.^292–294^ The JAK/STAT pathway, as a cytokine-mediated signal transduction pathway, can exacerbate disease development.^294^ For instance, IL-4 has a critical role in the pathogeny of atopic dermatitis. Moreover, JAK1 and JAK3 are related to Th1 cell activation in the acute phase of atopic dermatitis.^295^ Multiple studies have shown that STAT6 exerts a significant effect on the immune response by regulating B-cell differentiation and contributing to IgE class switching.^296^ Therefore, STAT6 is a potent transductor and activator in allergic disorders. Abnormalities in Th2 immune responses are also associated with high JAK/STAT pathway activity. Moreover, they cause an increase in cytokine, chemokine, and IgE production leading to the exacerbated inflammatory reactions of atopic dermatitis.^297^

#### Rheumatoid arthritis

Rheumatoid arthritis (RA) is a complex and chronic systemic inflammatory disease involving multiple organs and tissues that most frequently affects diarthrodial joints.^298,299^ Although many novel therapeutic approaches have been developed through a deeper understanding of the molecular and cellular mechanisms of rheumatoid arthritis, a series of problems remain to be resolved, including inadequate or partial responses, a lack of appropriate biomarkers, and drug-related toxicity. Cytokines have been reported to accelerate RA progression, as evidenced by a significant increase in proinflammatory cytokines, such as TNF-α, IL-1ß, IL-6, and IFN-γ. Some of these cytokines exert a profound influence on RA primarily by the JAK/STAT pathway. For example, IL-6 and IFN-γ can mediate the activation of the JAK/STAT pathway. In addition, TNF is able to activate this pathway by causing STAT3 phosphorylation.^300,301^ Besides, STAT4 and STAT6 polymorphisms play central roles in RA.^129,302^

### Other diseases

#### Parkinson’s disease

Parkinson’s disease (PD) is an age-related disease that manifests as significantly dysfunctional dopaminergic neurons and accumulation of misfolded α-synuclein, leading to severe disease burdens for older adults. Symptoms of PD include movement disorders and poor memory. Oligomeric α-SYN produced by neurons can activate microglia and macrophages and generate proinflammatory meditators.^303–305^ Various data obtained from PD patients and animal models indicate that the toxic effect of α-SYN in neurons is potentially exacerbated via neuroinflammatory processes. Thus, inflammation may contribute to the pathogenesis and development of PD.^306^ Recent studies showed that aberrant expression of α-SYN was associated with the activation of the JAK/STAT pathway, resulting in dysfunction of innate and adaptive immune responses and ultimately inducing neurodegeneration. The increase in the number of microglia and macrophages plays an important role in the progression of PD. MHC Class 2 is considered a biomarker that can be used to assess the activation of microglia and macrophages and the expression of a gene induced by STAT activation. α-SYN is involved in the regulation of proinflammatory markers, including MHC Class 2, TNF, inducible nitric oxide synthase (iNOS), IL-6, and CCL2 in myeloid tissue from the midbrain.^307,308^ The JAK/STAT pathway is required for the transmission of signals induced by a large number of cytokines/chemokines. T cells and myeloid cells may be activated and polarized into pathogenic phenotypes when the JAK/STAT pathway is aberrantly activated.^8,309^ Importantly, preclinical data obtained from PD rat models confirmed that JAK inhibitors could significantly repress inflammatory cytokine production. In general, suppression of the activated JAK/STAT pathway may be a promising therapeutic target for treating patients with PD. Myeloid cells are reported as a significant risk factor related to the pathogenesis of PD.^308,310^ Suppressing the JAK/STAT pathway blocks myeloid cell transformation into a proinflammatory phenotype, which indirectly dampens innate immune reactions in the midbrain. IFN-γ and IL-6 are known as the most potent activators, and enhanced levels of both IFN-gamma and IL-6 have been found in patients with PD.^311–313^ JAK inhibitors can reduce IL-6 or IFN-γ serum levels in patients. These results indicate that patients can acquire clinical benefits when activation of the JAK/STAT pathway is inhibited.

#### Hair loss

A typical growth cycle of hair consists of three phases: (1) the growth phase (anagen), (2) the regression phase (catagen), and (3) the rest phase (telogen).^314^ Hair loss upon entry into the growth phase leads to complications. Several factors potentially contribute to disease pathogenesis, including hair follicle miniaturization and immune dysregulation. Recent evidence has shown that small-molecule inhibitors, such as JAK inhibitors, are effective for treating hair growth disorders. Activation of the JAK/STAT pathway can drive hair follicles into a quiescent state,^315^ leading to decreased hair growth capacity. Suppression of the JAK/STAT signaling pathway induces hair follicle exit from a resting state and enhances the ability of hair follicles to enter the hair cycle. Hyperactive JAK/STAT signaling in aged mice is closely associated with the suppression of hair follicle stem cell function in vitro.^316^ Furthermore, phosphorylated STAT5 plays a central role in regulating HF stem cells during pregnancy.^317^ Due to the importance of the dermal papilla (DP) in continual hair follicle cycling,^318^ various signaling pathways in the DP have been uncovered. STAT5a and STAT5b are significantly upregulated in the DP,^319^ while knockout of STAT5b in mice results in an apparently delayed entry to anagen in the early time of postnatal hair follicle growth.^320^ Moreover, previous studies have identified activated STAT5 as a vital switch to drive natural growth when post-developmental hair follicle cycle begins in mesenchymal cells.^321^ However, many functional experiments have also demonstrated that the JAK/STAT pathway may have an opposite role in the hair cycle compared with protective effects. Inhibitors blocking JAK/STAT signaling are shown to be effective for hair growth by inducing growth in the resting hair follicle.^322^ Various molecular mechanisms of the JAK/STAT signaling pathway have been discovered in different stages of hair growth. OSM is a negative regulator involved in hair growth as it can activate the JAK-STAT5 pathway to maintain the hair follicles in a static state.^323^ Data from these studies reveal the action of the JAK/STAT pathway in different conditions is not always toward a beneficial or harmful direction, thus more additive investigations need to be performed on these mechanisms. Moreover, IL-6 has a higher expression in keratinocytes, which links with the suppression of hair growth.^324^

#### Age-related diseases

Chronic inflammation is a typical symptom in aging and age-related diseases.^325^ The increased production of proinflammatory cytokines and chemokines is a major risk factor for many age-related diseases and cellular senescence.^326,327^ The cooperative effect of senescent cells and inflammation significantly contributes to age-related pathology. The JAK/STAT pathway is a common cytokine-medicated cascade and important for cytokine production.^201,328^ Senescent cells accumulating in adipose tissue appear to be the development of a senescence-associated secretory phenotype (SASP), which is closely associated with higher activation of the JAK/STAT pathway and inflammation. Preclinical studies have demonstrated that JAK1 and JAK2 activation in the adipose tissue of old rats was increased. STAT3 plays a central role in inducing and maintaining an inflammatory microenvironment by mediating a wide range of SASP components, including IL-6, IL-8, plasminogen activator inhibitor 1, monocyte chemoattractant protein-1 (MCP-1), and GM-CSF.^201,329^ These results indicate that JAK1 and JAK2 regulate the effects of the SASP. A significant reduction in activated STAT3 upon treatment with a JAK inhibitor was observed, suggesting a specific interaction between STAT3 and age-related adipose tissue inflammation. Increased IL-6 in serum is linked with low physical activity and frailty in older adults. A number of methods have been used to delay aging, such as GH receptor knockout (GHRKO) and surgical removal of visceral fat.^330,331^ The gene disruption of GH receptor in mice results in a longer life span and less or delayed occurrence of age-related or malignant diseases potentially via inducing metabolic changes and increasing insulin sensitivity, indicating that insulin signaling exerts an important impact on aging.^330^ It is reported that JAK2 activity is controlled by the interaction between insulin signaling and angiotensin II (AII) systems.^332^ In response to insulin-induced stimulation, JAK2 is phosphorylated and forms a complex with STAT1, named JAK2-STAT1 complex, to transmit directly insulin signal towards the nucleus.^333^ As GHRKO mice do not respond to GH, this is accompanied by severely reduced plasma levels of insulin-like growth factor-1 (IGF-1), suggesting IGF-1 may be involved in aging. IGF-1 can act as a growth factor through JAK/STAT pathway in many tissues, for example, IGF-1 selectively activates JAK1 but not JAK2 or TYK2. However, few studies have been performed to identify the molecular mechanism JAK/STAT pathway regulating age-related diseases. This potentially is an important insight to extend life span.

## Inhibitors and clinical applications

**Fig. 5:**
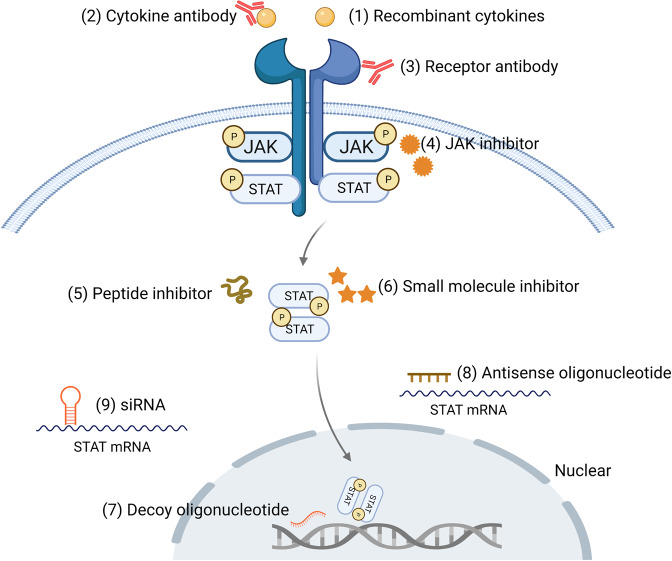
Therapeutic targets of the JAK/STAT signaling pathway. (1) Recombinant cytokines, (2) cytokine antibodies, and (3) receptor antibodies are designed to target cytokines or receptors; (4) JAK inhibitors are designed to target JAKs; and (5) peptide inhibitors, (6) small-molecule inhibitors, (7) decoy oligonucleotides (ODNs), (8) antisense oligonucleotides (ASOs), and (9) siRNAs target STATs. Created with BioRender.com

Medications that target the JAK/STAT pathway can be classified into three types: (1) cytokine or receptor antibodies, (2) JAK inhibitors, (3) STAT inhibitors (Fig. 5). They have been applied to various cancers and autoimmune diseases. Several of these drugs have been approved for the market, such as the JAK inhibitors: tofacitinib and ruxolitinib. Most of these drugs are in preclinical and clinical trials. We will introduce them in this section.

### Manipulation of activators

Upstream cytokines and receptors are crucial for determining the functions of the JAK/STAT signaling pathway. Thus, manipulating upstream activators has long been used in clinical settings, and such manipulation is performed with recombinant cytokines, cytokine–antibody fusions, and cytokine/receptor-blocking antibodies.

#### Engineered cytokines

As we previously discussed, a large majority of cytokines are involved in the JAK/STAT signaling pathway. Their enhancement or blockade has been applied to treat multiple diseases. For example, recombinant IL-2 is approved to treat metastatic melanoma and renal cell carcinoma, type 1 interferons are used for the treatment of Hepatitis C virus, IFNα is used for treating the viral infections, and TNF is a treatment of cancer. Research on other interleukins is ongoing. Nevertheless, adverse events and low selectivity have restrained their clinical use. Engineered cytokines such as ALKS 4320 (recombinant IL-2) and RMP16 (recombinant TNF) have been tested in preclinical studies and demonstrated relatively better efficacy and less toxicity.^334,335^

Cytokine–antibody fusions are novel biopharmaceuticals that increase the therapeutic index of cytokine “payloads”. They are generated by the fusion of two compounds. One of the compounds is an antibody specific to accessible markers, which can be overexpressed in certain diseases. Another is therapeutic payloads, including cytokines, these fusion proteins can dramatically increase the therapeutic index of the cytokine payload. These include ch14.18-IL-2, Hu14.18-IL-2, NHS-IL2LT, DI-Leu16-IL-2, BC1-IL-12, L19-TNF.^336^

#### Antibody-based blockade

Antibody-based blockade of cytokines and their cognate receptors has been extensively studied. For example, blockade of IL-2, IL-12, IL-17, and TNF has been successfully used to treat chronic inflammatory diseases such as RA, IBD, and psoriasis. Some of these blockades are market-approved, such as anti-IL-2Rα, anti-IL-5, anti-IL-6, anti-IL-6R, anti-IL-12, and anti-IL-23. The anti- IL-2Rα antibody, also known as daclizumab, markedly inhibited the phosphorylation of JAK1, JAK3, and STAT5a/b, thus significantly decreasing transplant rejection.^337^ Siltuximab is an IL-6 antagonist and has been approved for the treatment of idiopathic multicentric Castleman’s disease (iMCD). Tocilizumab, an anti-IL-6R humanized antibody, has been approved for the treatment of RA, cytokine release syndrome (CRS), and iMCD. New-generation anti-IL-6 and anti-IL-6R monoclonal antibodies improved binding affinity and specificity and reduced toxicity. They are in clinical trials focused on various diseases. For example, sarilumab, sirukumab, clazakizumab, and olokizumab target IL-6, vobarilizumab, olamkicept, satralizumab, and NI-1202 target IL-6R.^338,339^

IL-5 is important for the priming and survival of mature eosinophils, and it is crucial for the proliferation and maturation of eosinophil progenitors. Anti-IL-5 antibodies are used in diseases such as eosinophilic asthma, eosinophilic oesophagitis, hypereosinophilic syndrome, and eosinophilic granulomatosis with polyangiitis (EGPA).^340^ Mepolizumab and reslizumab act against IL-5, and benralizumab targets IL-5R. In EGPA patients, mepolizumab combined with standard treatment led to prolonged remission and less steroid use.^341^ IL-12 and IL-23 share the signature p40 subunit, and an anti-IL-12/23 p40 antibody (p40 mAb) interferes with Tfh cell differentiation and inhibits proinflammatory cytokine secretion. Thus, this antibody attenuates chronic graft-versus-host disease in murine models of lupus nephritis.^342^ P40 mAb has been tested in many diseases in preclinical studies, such as psoriasis and Crohn’s disease.^343,344^ The results of phase 3 clinical trial including 312 adults with active psoriatic arthritis (PsA) show that ustekinumab (45/90 mg q12 weeks) led to significant and long-term improvement of symptoms/signs, including in patients who had previously received anti-TNF treatment.^345^ Pegvisomant, a competitive GH receptor antagonist, is used to treat acromegaly as monotherapy or combinational therapy with somatostatin analogs. Pegvisomant is generally used as a second-line therapy and has a high curative effect but the high cost in the treatment of acromegaly.^346^

More cytokine/receptor antibodies are being studied, such as anti-IL-4, anti-IL-4R, anti-IL-5R, anti-IL-6R, anti-IL-9, and anti-IL-13.^6^

### JAK inhibitors

**Fig. 6:**
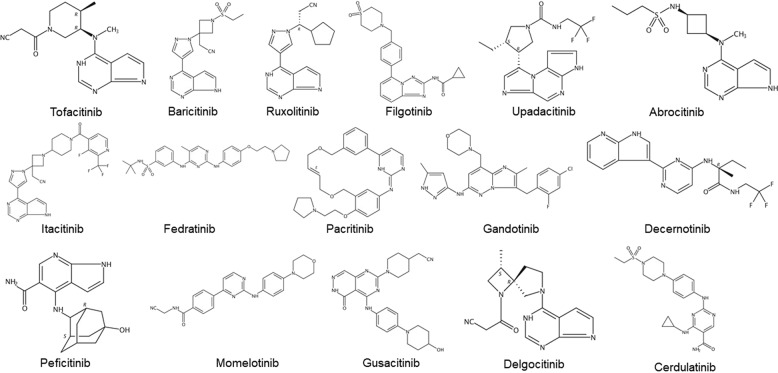
Chemical structures of JAK inhibitors.

JAK inhibitors are a group of small-molecule inhibitors with different chemical structures (Fig. 6). The therapeutic effects of JAK inhibitors are based on two factors. First, JAKs mediate various cellular activities. Inhibition of JAK function can cause immunosuppression and decrease the abnormally elevated serum proinflammatory cytokines mediated by the JAK/STAT signaling pathway. Second, in some diseases, such as in myeloproliferative diseases and cancers, identifying gain-of-function JAK mutants enables treatment through their inhibition.^3^ Various JAK inhibitors are being studied in preclinical and clinical studies. Tofacitinib and baricitinib are the first orally available JAK inhibitors to be approved in treating RA and other autoimmune diseases.^347^

Serious adverse events are always a concern when JAK inhibitors are considered for treatment. All JAK inhibitors have similar adverse effects, including infection, hyperlipidemia, and cytopenia. The first two JAK inhibitors approved for RA treatment, tofacitinib and baricitinib, have black box warnings of severe infections and malignancies. Some preclinical studies indicated that a reduction in lymphocytes, NK cells, and neutrophils might be associated with biological differences in different subtypes of JAK inhibitors.^348^ In addition to clinical applications, JAK inhibitors can be powerful tools for scientific research. For example, events downstream of certain ligands have been investigated and mechanisms of immune checkpoint blockade drug resistance have been delineated.

The first-generation JAK inhibitors (tofacitinib, oclacitinib, baricitinib, and ruxolitinib) are adenosine triphosphate (ATP)-competitive compounds. They target the JAK homology 1 tyrosine kinase domain in its active conformation. The ATP-binding pocket structure is highly conserved. Thus, first-generation JAK inhibitors target more than one JAK member.^30^ Most next-generation JAK inhibitors are also ATP-competitive. Nevertheless, there are also some JAK inhibitors (such as Deucravacitinib) that target the JH2 domain of JAK (Table 4).^349^

**Table 4:** Clinical application of JAK inhibitors

Drug	Selectivity	FDA/EU-approved Indications	Indications under phase II/III clinical trials	Reported adverse events
Tofacitinib (Xeljanz and Tasocitinib)	JAK1, JAK3	(1) RA (2) Ulcerative colitis (3) Polyarticular juvenile idiopathic arthritis	(1) Crohn’s disease (2) Alopecia areata (3) Dermatomyositis (4) Atopic dermatitis (5) COVID-19 (6) Psoriasis (7) Ankylosing spondylitis	(1) Infections (2) Gastrointestinal perforation (3) Thromboembolism
Baricitinib (LY3009104, INCB028050, and Olumiant)	JAK1, JAK2	(1) RA	(1) Lupus (2) Atopic dermatitis (3) COVID-19 (4) Alopecia areata	(1) Infections (2) Hyperlipidaemia (3) VTE
Ruxolitinib (Jakafi, INCB18424, INCB018424, and Jakavi)	JAK1, JAK2	(1) Myelofibrosis (2) aGVHD (3) Polycythaemia vera	(1) Alopecia areata (2) Vitiligo (3) Essential thrombocythemia (ET) (4) COVID-19 (5) Atopic dermatitis	(1) Anemia (2) Thrombocytopenia (3) Neutropenia (4) Hypokalemia (5) Infections (6) Peripheral edema
Filgotinib	JAK1	(1) RA (EU approved)	(1) IBD (2) PsA (3) Ankylosing spondylitis	(1) Nasopharyngitis (2) Headache (3) Infections
Upadacitinib (RINVOQ)	JAK1	(1) RA	(1) Crohn’s disease (2) Ulcerative colitis (3) Aatopic dermatitis (4) PsA (5) Ankylosing spondylitis	(1) Infections (2) Malignancies (3) Elevated lipid parameters, creatine phosphokinase, and hepatic aminotransferase, (4) Low blood cell counts (5) Stroke (6) Venous thromboembolisms
Abrocitinib	JAK1	/	(1) Aatopic dermatitis	(1) Headache (2) Diarrhea (3) Nausea (4) Upper respiratory tract infection (5) Hematologic abnormalities (6) Nasopharyngitis
Itacitinib (INCB039110)	JAK1	/	(1) Arthritis (2) IBD (3) aGVHD (4) CRS during CAR-T therapy (5) B-cell lymphoma	(1) Diarrhea (2) Anemia (3) Pneumonia (4) Pyrexia (5) Elevated live enzymes
Fedratinib (Inrebic)	JAK2	(1) Myelofibrosis	(1) AML	(1) Wernicke’s encephalopathy (2) Anemia (3) Thrombocytopenia (4) Diarrhea (5) Nausea (6) Vomiting (7) Abnormal liver and pancreatic tests
Pacritinib	JAK2	/	(1) Myelofibrosis (2) AML	(1) Anemia (2) Thrombocytopenia (3) Diarrhea (4) Cardiac failure (5) Pyrexia (6) Pneumonia
Gandotinib (LY2784544)	JAK2	/	(1) Myeloproliferative neoplasms (MPN) (2) Polycythemia vera (3) Essential Thrombocythemia	(1) Anemia (2) Hyperuricaemia (3) Fatigue (4) Diarrhea (5) Thrombocytopenia
Decernotinib	JAK3	/	(1) RA	(1) Neutropenia (2) Lymphopenia (3) Hyperlipidaemia (4) Elevated hepatic transaminases
Peficitinib (Smyraf, ASP015K, and JNJ-54781532)	JAK3	/	(1) RA (2) Psoriasis (3) Ulcerative Colitis	(1) Infections (2) Plasma CK increase (3) Lymphopenia (4) Gastrointestinal perforation (5) Sepsis
Momelotinib (CYT387)	JAK1, JAK2	/	(1) Myelofibrosis	(1) Anemia (2) Neutropenia (3) Thrombocytopenia (4) Liver/pancreatic test abnormalities (5) Treatment-emergent peripheral neuropathy (TE-PN) (6) Diarrhea (7) Cough (8) Nausea
Gusacitinib	JAK1, JAK2, JAK3, TYK2	/	(1) Atopic dermatitis	(1) Hyperlipidaemia (2) Hypertension
Delgocitinib	JAK1, JAK2, JAK3, TYK2	(1) Atopic dermatitis (Japan)	(1) Alopecia areata (2) Chronic hand eczema (3) Lupus erythematosus	(1) Nasopharyngitis (2) Contact dermatitis (3) Acne
Cerdulatinib	JAK1, JAK2, TYK2	/	(1) Non-Hodgkin lymphoma	(1) Anemia (2) Fatigue (3) Diarrhea

*RA* rheumatoid arthritis, *COVID-19* coronavirus disease 2019, *VTE* venous thromboembolism, *aGVHD* acute graft-versus-host disease, *IBD* inflammatory bowel disease, *PsA* active psoriatic arthritis, *AML* acute myeloid leukemia

#### First-generation JAK inhibitors

##### Tofacitinib

Tofacitinib, also named Xeljanz or CP690, 550, was the first JAK inhibitor studied in humans. Tofacitinib preferentially inhibits JAK1 and JAK3 and, to a lesser extent, JAK2 and TYK2. It is the first JAK inhibitor approved mainly to treat RA and other autoimmune diseases. Tofacitinib blocks the γc cytokine-receptor signaling pathway through JAK1 and JAK3 in T cells. Thus, it interferes with Th1 and Th2 differentiation and impairs the production of inflammatory Th17 cells. Tofacitinib also suppresses cytokine production through both innate and adaptive processes, including common γ chain cytokines IFN-γ, TNF, IL-6, IL-12, IL-17, and IL-23. Nevertheless, tofacitinib increased serum levels of IL-35 and IL-35 might be an indicator of the disease activity attenuated by tofacitinib efficacy.^350,351^

Tofacitinib is effective in preclinical studies and has been applied in various phase 2 and phase 3 clinical trials. Most often, it is applied to patients whose previous therapies failed. Tofacitinib is under investigation for use in various diseases, including RA, ulcerative colitis, Crohn’s disease, relapsing polychondritis, atopic dermatitis, alopecia areata, cutaneous dermatomyositis, psoriasis, psoriatic arthritis, and ankylosing spondylitis.^352–360^ In total, 5 or 10 mg of tofacitinib twice a day is the most commonly used dosage.^352^ Recently, tofacitinib was regarded as a candidate in treating coronavirus disease 2019 (COVID-19), although no published study showed the benefits, several clinical trials are ongoing, clinical trial identifiers, including NCT04415151, NCT04469114, NCT04390061, and NCT04332042.^361^

Adverse events of tofacitinib are mostly tolerable, including opportunistic infections (OIs), gastrointestinal perforation, thromboembolism, and herpes zoster.^362,363^ Tuberculosis (TB) was the most common OI reported thus far.^364^ Incidence rates of thromboembolic events were elevated in patients with baseline cardiovascular or venous thromboembolism (VTE) risk factors versus without these risk factors, indicating that VTE is an important risk factor for thromboembolism during tofacitinib treatment.^365^ For patients with chronic viral infections such as HBV infection, tofacitinib therapy appears safe, but physicians should be aware of the risk of HBV reactivation.^366^ For pregnant patients, unintentional exposure to tofacitinib does not appear to be associated with an increased risk to the fetus.^367^ Twenty-four weeks of tofacitinib treatment appeared to be associated with a lower risk of future major adverse cardiovascular events (MACE) risk, including myocardial infarction, stroke, cardiovascular death.^368^ Based on the limited data, treatment of RA with tofacitinib does not increase the incidence of malignant tumors.^369^ Nevertheless, more safety data are needed as tofacitinib inhibited several key cytokines and immune cells.

##### Baricitinib

Baricitinib is a selective oral JAK1 and JAK2 inhibitor with moderate activity against TYK2 and significantly less activity against JAK3. It is generated by modifying the structure of tofacitinib.^347^ Baricitinib achieved efficacy in multiple autoimmune diseases, including RA, lupus erythematosus, juvenile dermatomyositis, atopic dermatitis, and IFN-mediated autoinflammatory disease, it inhibits the worsening of symptoms and reduces inflammation. Similar to tofacitinib, baricitinib is mostly used in patients whose previous therapies failed, 2 or 4 mg once daily is recommended in most randomized controlled trials (RCTs).^370–377^

Moreover, baricitinib decreased albuminuria over 24 weeks of treatment in patients with diabetes and diabetic kidney disease (DKD). A possible explanation is that baricitinib treatment decreased inflammatory biomarkers associated with DKD pathophysiology, including CCL2 (MCP-1), CXCL10 (IP-10), SSA, tumor necrosis factor receptor (STNFR)1 and STNFR2, VCAM1, and intercellular adhesion molecule-1 (ICAM-1).^378^ Baricitinib has also been studied in many animal models to investigate its potential for broader clinical application, such as in human immunodeficiency virus (HIV)-associated neurocognitive disorders and osteoporosis.^379,380^

More importantly, baricitinib is predicted to be effective in the treatment of COVID-19. AP2-associated protein kinase 1 (AAK1) is one of the known regulators of endocytosis. Disruption of AAK1 might interrupt the virus from getting into lung AT2 alveolar epithelial cells via ACE2 receptor. Baricitinib is one of the six high-affinity AAK1-binding drugs, which also binds cyclin G-associated kinase, another regulator of endocytosis. Moreover, baricitinib inhibits inflammation via JAK1/2-mediated cytokine signaling.^381,382^ One of the most important effects was the rapid and remarkable inhibition of macrophage production of the cytokines and chemokines critical for inflammation and neutrophil recruitment.^383^ A double-blind, randomized, placebo-controlled trial including 1033 hospitalized adults revealed that baricitinib plus remdesivir was superior to remdesivir alone in reducing recovery time and accelerating improvement in clinical status.^384^ Patients with severe COVID-19 had a significant reduction in serum IL-6, IL-1β, TNF, increased antibodies against the SARS-CoV-2 spike protein, and rapid recovery of B-cell and T-cell frequencies.^385^

With exposure as many as 5.5 years, baricitinib has an acceptable safety profile. There is no difference in serious adverse effects such as death, adverse events leading to drug discontinuation, MACE, and malignancies.^386^ The most frequently reported AE was dose-dependent increased low-density lipoprotein (42.1%), followed by an increased risk of infections, including herpes zoster and TB. Baricitinib should be used with caution in patients with VTE risk factors.^387^

##### Oclacitinib

Oclacitinib is a cyclohexylamino pyrrolo [2,3-d] pyrimidine derivative that targets the JAK family. It is the most potent in inhibiting JAK1. Oclacitinib is currently used mostly to treat canine and cat pruritus and allergic skin diseases. There is no report of this drug being used to treat humans.^388,389^

##### Ruxolitinib

Ruxolitinib, also named INCB018424 or INC424, was found to inhibit JAK1 and JAK2, which is often dysregulated in myelopathies. Ruxolitinib is oral or topical administered. Clinical studies of ruxolitinib for the treatment of malignant tumors, acute graft-versus-host disease (aGVHD), MF, polycythaemia vera, alopecia areata, vitiligo, essential thrombocythemia, and COVID-19 are conducted worldwide.^390–397^ Ruxolitinib was first approved for the treatment of MF by the US FDA in 2011 and approved by the European Medicines Agency in 2012, followed by approval for the treatment of polycythaemia vera in 2014.^398^ Although ruxolitinib achieved clinical benefits in many patients with autoimmune diseases, it failed to significantly improve overall survival in patients with malignant tumors, including pancreatic cancer and colorectal cancer.^390,399,400^ Ruxolitinib has received much attention in the past year for its efficacy in treating COVID-19.^396^ Although there was no significant difference between ruxolitinib plus standard-of-care treatment and placebo, ruxolitinib improved the clinical symptoms and chest computed tomography images in COVID-19 patients.^396^

In 2011, ruxolitinib was the first drug approved by the US FDA to treat patients with intermediate or high-risk MF. According to previous clinical trials, the starting dose of ruxolitinib is 20 mg taken orally twice daily for patients with platelet counts higher than 200 × 10^9^/L, and 15 mg twice daily for those with a platelet count between 100 × 10^9^/L and 200 × 10^9^/L. The dose was increased based on the response and a maximum of 25 mg was recommended twice daily. Ruxolitinib is not specific for the JAK2^V617F^ mutation, and its efficacy in MF is mainly due to the attenuation of the constitutive activation of the JAK/STAT pathway and myelosuppression.^398^ For ruxolitinib-resistant or ruxolitinib-intolerant MF patients, another JAK2-selective inhibitor fedratinib might lead to clinical benefits and alleviate adverse events.^401^ However, another JAK1 and JAK2 inhibitor, momelotinib, failed to provide better clinical benefits for patients previously treated with ruxolitinib.^402^

The most common toxicity induced by ruxolitinib is myelosuppression, which results in anemia (64.8%), thrombocytopenia (62.0%), and neutropenia (47.9%). Other common adverse events include hypokalaemia (49.3%), peripheral edema (45.1%), and a high treatment discontinuation rate.^391^ The high treatment discontinuation rate is primarily caused by clinical benefit loss and drug toxicity. It has also been reported that severe withdrawal symptoms occur during MF treatment called “ruxolitinib discontinuation syndrome”. It is characterized by the acute relapse of disease symptoms and worsening splenomegaly.^403^ These events are related to the acute rebound of cytokine storms. Careful tapering is regarded as a preventive strategy.^404^ When ruxolitinib was used to treat skin depigmentation of vitiligo, adverse events also included application site pruritus and acne.^405^ Importantly, because JAK inhibitors interfere with many physical and pathological processes, ruxolitinib may be associated with immunosuppressive diseases such as severe and fatal infections, viral reactivation, and even Kaposi sarcoma (KS). KS was reported in an essential thrombocythemia patient. KS regressed 10 months after ruxolitinib discontinuation.^406,407^ When ruxolitinib was used to treat COVID-19, there were reports of severe purpuric lesions on the skin of dorsal and upper limbs, with a concomitant decrease in platelet counts. One patient displayed diffuse erythematous.^408^

#### Next-generation JAK inhibitors

JAK1 and JAK2 are critical for signal transduction by numerous cytokines, while JAK3 and TYK2 are activated by relatively few cytokines. Next-generation JAK inhibitors with higher specificity may reduce adverse events.

##### JAK1 inhibitors

*Filgotinib*: Filgotinib is a JAK1 inhibitor. Filgotinib inhibited Th1, Th2, and Th17 differentiation, and JAK1-dependent cytokines in a dose-dependent manner, including IL-2, IL-4, and IL-6, which plays vital pathological roles in chronic inflammation and autoimmune disorders.^409^ Filgotinib is mostly used for inflammatory and autoimmune diseases, including inflammatory bowel diseases, rheumatoid arthritis, PsA, and ankylosing spondylitis.^410–412^ In September 2020, the EU approved filgotinib for the treatment of moderate-to-severe RA patients who inadequately respond to one or more DMARD. The recommended dose for adults is 200 mg taken once daily. The same dose has been applied in a phase 2 clinical trial of moderate-to-severe Crohn’s disease, in which it led to significant clinical remission.^413,414^ More importantly, by combining high-throughput drug screening and the transcriptome analysis (differential analysis, gene set enrichment, and exon-intron landscape analysis), researchers found that filgotinib is not merely a JAK inhibitor, it can act as a splicing inhibitor and modulate HIV splicing, as well as inhibit T-cell activation, thus suppressing HIV-1 transcription and reducing the proliferation of HIV-infected cells. Therefore, filgotinib may be a candidate drug for use in the therapy of acquired immunodeficiency syndrome patients.^415^

The most common adverse events are nasopharyngitis, headache, and upper respiratory infections. There were no reports of opportunistic infections, malignancy, gastrointestinal perforation, or death. More long-term safety data are needed for this new drug.

*Upadacitinib:* Upadacitinib, also named ABT-494, is an orally administered JAK1 inhibitor. It potently inhibits JAK1-dependent cytokines, including IL-6, OSM, IL-2, and IFNγ. On 16 August 2019, upadacitinib was approved to treat moderate to severely active RA in patients with inadequate response or intolerance to methotrexate. The recommended dosage is 15 mg taken once daily. 30 mg taken once daily provided only a small incremental benefit. Upadacitinib can be administered alone or in combination with methotrexate or DMARDs.^416^ Analysis of RA patient plasma proteins suggested that treatment with upadacitinib normalizes key pathways associated with RA pathobiology, including IL-1, IL-6, IFNγ, and TNF. Upadacitinib is also associated with leukocyte activity, including cell migration and inflammatory responses.^417^ Compared to the first approved JAK inhibitor, tofacitinib combined with methotrexate, upadacitinib displays better outcomes as both a monotherapy and a combination therapy at 3 and 6 months.^418^ In addition to its use as an RA treatment, researchers are exploring other indications of upadacitinib, such as Crohn’s disease, ulcerative colitis, atopic dermatitis, psoriatic arthritis, and ankylosing spondylitis.^419–423^

The most common adverse events are infections and increases in lipid parameters, creatine phosphokinase, and hepatic aminotransferase, followed by a reduction in neutrophil and lymphocyte counts. Serious adverse events, including death, stroke, and venous thromboembolic, were rare but reported in a phase 3 clinical trial with RA patients. More extensive and longer clinical trials are required to verify the safety of upadacitinib.^424^

*Abrocitinib*: Abrocitinib, also named PF-04965842, is an oral JAK1 inhibitor. Abrocitinib is mainly used to treat atopic dermatitis. Phase1, 2, 3 clinical trials reported the clinical efficacy and acceptable tolerability, but no obvious improvements were observed between abrocitinib and dupilumab, a monoclonal antibody targeting IL-4Rα.^425,426^

There were no deaths or serious adverse events reported. Headache, diarrhea, nausea, upper respiratory tract infection, hematologic abnormalities, and nasopharyngitis are the most common adverse events.^425^

*Itacitinib*: Itacitinib (INCB039110) is a selective JAK1 inhibitor that has exhibited efficacy in preclinical studies of arthritis, IBD, and aGVHD.^427^ Moreover, itacitinib dose-dependently reduced the levels of multiple cytokines common to CRS during CAR-T therapy. Thus, itacitinib can be a prophylactic agent for CAR-T therapy-induced CRS, and a relative phase 2 clinical trial (NCT04071366) is ongoing.^428^ The commonly used dosage in clinical trials is 200 mg or 300 mg taken once daily, and phase 1 clinical trials preliminarily demonstrated the safety and efficacy of itacitinib. Larger-scale clinical trials are required in the future.^429^

##### JAK2 inhibitors

*Fedratinib*: Fedratinib is an orally administered kinase inhibitor that selectively targets both wild-type and mutated JAK2 and FMS-like tyrosine kinase 3 (FLT3), and inhibits the phosphorylation of STAT3 and STAT5. Fedratinib received approval on 16 August 2019 in the USA for the treatment of patients with intermediate- or high-risk primary or secondary MF. The recommended dosage is 400 mg taken once daily in patients with platelet counts of more than 50 × 10^9^/L. The dosage should be one-half the recommended dose in patients with severe renal impairment or patients concomitantly receiving potent CYP3A4 inhibitors. Fedratinib prolonged survival in many murine tumor models, including prostate cancer. However, the development of fedratinib for use in treating malignant tumors has been discontinued.^430^

Adverse events warnings include severe to fatal encephalopathies, such as Wernicke’s encephalopathy. A putative mechanism for this adverse effect is related to the individual human thiamine transporter, which is inhibited by fedratinib. Fedratinib mediates the thiamine uptake in Caco-2 cells, and Wernicke’s encephalopathy is mediated by thiamine deficiency. Inhibition of thiamine uptake seems to be unique to fedratinib and is not a feature of other marketed JAK inhibitors.^431^

*Pacritinib*: Pacritinib inhibits both JAK2 and FLT3. Moreover, it has high selectivity against the JAK2^V617F^ and FLT3 D835Y mutants, which are frequently found in MPN and AML. Pacritinib inhibits IRAK1 (an IL-1 receptor kinase), and IRAK1 is commonly mutated in two dysregulated hematopoiesis diseases (myelodysplastic syndromes and Fanconi anemia).^432^ Pacritinib is currently mainly used in MF and AML patients with a dosage of 200 mg twice a day or 400 mg once a day.^433^ In patients with MF and thrombocytopenia, 200 mg of pacritinib twice daily is better than 400 mg of pacritinib once daily in terms of hemoglobin and reduction in transfusion burden. Moreover, pacritinib is superior to the best available therapy (BAT), including ruxolitinib, in reducing spleen volume and symptoms.^434^ More importantly, pacritinib is effective at all JAK2^V617F^ allele burden quartiles and in JAK2^V617F^-negative MF patients, suggesting that pacritinib may be uniquely suited for treating myelodepletive MF patients.^435^ Pacritinib has also been researched for treating several other types of cancers, including colon, rectal, and non-small cell lung cancer, but no objective response was observed in colorectal cancer.^436,437^

The most common grade 3/4 adverse events were anemia, thrombocytopenia, and diarrhea, and the most severe adverse events were anemia (5%), cardiac failure (2%), pyrexia (2%), and pneumonia (2%). Twenty-seven (12%) patients died due to serious adverse events in the pacritinib group compared with 14 (13%) in the BAT group.^433^

*Lestaurtinib*: Lestaurtinib is a multi-kinase inhibitor that targets a broad array of kinases, including JAK2, FLT3, RET, and TRK. However, lestaurtinib more effectively inhibits FLT3 than other kinases.^438^

*Gandotinib*: Gandotinib, also named LY2784544, is another JAK2 inhibitor. It inhibits JAK2^V617F^ mutant in a dose-dependent manner and may inhibit additional JAK2 mutants in preclinical studies. Gandotinib is used to treat MPN, polycythemia vera, and essential thrombocythemia in phase 1/2 clinical trials. It demonstrated acceptable toxicity and a maximum tolerated dose of 120 mg taken daily.^439,440^

##### JAK3 inhibitors

*Decernotinib*: Decernotinib is a newly developed JAK inhibitor that potently inhibits JAK3 with limited or no measurable potency against the other three JAKs or non-JAK kinases. Decernotinib is effective in animal models in reducing ankle swelling T-cell-mediated inflammatory response in the skin.^441^ Three phase 2 clinical trials demonstrated that decernotinib can reduce the signs and symptoms of RA patients when it was administered monotherapy or in combination with DMARD or methotrexate.^442–444^ Adverse events include neutropenia, lymphopenia, hyperlipidaemia, and elevated hepatic transaminases. Lymphopenia may be associated with JAK3-associated cytokines, including IL-7 and IL-15.^445^ More clinical data are needed to verify the efficacy and safety of decernotinib treatment of more diseases.

*Peficitinib*: Peficitinib, also named Smyraf, ASP015K, and JNJ-54781532, is an orally administered JAK3-selective inhibitor. It inhibits IL-2-induced T-cell proliferation and STAT5 phosphorylation. Peficitinib was developed in Japan for the treatment of RA and received approval in Japan and Korea to treat RA patients inadequately responding to conventional therapies.^446^ The peficitinib pharmacokinetic profile is altered in subjects with moderate-to-severe hepatic impairment. Peficitinib exposure and adverse effects are similar to or without renal impairment.^447,448^ The recommended dosage is 150 or 100 mg once daily and 50 mg once daily for patients with moderate liver dysfunction. It is contraindicated in patients with severe liver dysfunction. Peficitinib is mainly investigated for treating RA. In addition to RA, peficitinib has been investigated for its efficacy in treating other autoimmune diseases, including psoriasis and ulcerative colitis.

The most frequent adverse events are nasopharyngitis, herpes zoster infection, a plasma creatine kinase increase, and lymphopenia, followed by pneumonia, pharyngitis, epipharyngitis, upper respiratory tract infection, bronchitis, influenza, and cystitis. The rare severe adverse events are gastrointestinal perforation and sepsis.^446^ Peficitinib does not have a significant effect on the pharmacokinetics of rosuvastatin, a statin.^449^

##### Pan-JAK inhibitors

*Momelotinib*: Momelotinib, formerly named CYT387, is an oral selective ATP-competitive inhibitor of JAK1, wild-type and mutated JAK2, and activin A receptor type 1.^450^ Momelotinib induced growth suppression and apoptosis in JAK2-dependent hematopoietic cell lines when added between 0.5 and 1.5 μM, without affecting nonhematopoietic cells. In murine models, momelotinib is unable to completely eliminate JAK2-dependent cells, and MPN often reappears, suggesting that it is not curative and is better used in combinational therapy.^451^ In clinical studies, Momelotinib is effective in treating MF patients at a dosage of 200 mg twice a day or 300 mg once daily. In the patients with the JAK2^V617F^ mutation, momelotinib significantly reduced the allele burden (21.1%).^452^ In a 7-year follow-up of 100 MF patients, momelotinib had been discontinued in 91% of patients after a median treatment of 1.4 years, suggesting that momelotinib is well-tolerated and induces long-term benefits. More importantly, in contrast to most other JAK2 inhibitors, momelotinib improved anemia in a substantial fraction of patients, which may be attributed to the inhibitory effects of momelotinib against ALK2-mediated hepcidin expression.^453^ In patients with previous ruxolitinib failure, momelotinib was not superior to the BAT in reducing spleen volume, which was reduced by 35% compared with the baseline volume. There is no evidence that JAK2 inhibitors are effective in reversing MF or inducing cytogenetic or molecular remission, and the efficacy of momelotinib contributes to the nonspecific inhibition of inflammatory cytokines.^402^ Momelotinib combined with trametinib does not perform better than single-agent trametinib in KRAS-mutated non-small cell lung cancer.^454^

The most frequent adverse events of momelotinib are diarrhea, cough, and nausea in patients with MF.^455^ Grade 3/4 adverse events include anemia, neutropenia, thrombocytopenia, and liver/pancreatic test abnormalities.^453,455^ A significant adverse event of momelotinib is treatment-emergent peripheral neuropathy (TE-PN), which has been documented with a 44% (44/100) incidence rate, and TE-PN is significantly associated with prolonged survival due to treatment response.^456^

*Gusacitinib*: Gusacitinib, also named ASN002, is a multi-target JAK inhibitor that targets JAK2, JAK3, TYK2, with a lesser extent inhibit JAK1. Gusacitinib also inhibits spleen tyrosine kinase (SYK). Both JAK and SYK are involved in the pathogenesis of atopic dermatitis. In two phase 1b clinical trials on atopic dermatitis, gusacitinib (40 mg or 80 mg daily) achieved efficacy rapidly and downregulated several biomarkers involved in systematic inflammation, such as E selectin.^457^ There were no serious adverse events that happened, and changes in serum cholesterol and blood pressure were observed.^458^

*Delgocitinib*: Delgocitinib, also named JTE-052, inhibits all for members of the JAK family. Delgocinib is developed in Japan for the treatment of autoimmune disorders and hypersensitivity. On 23 January 2020, topical delgocinib 0.5% ointment received its first approval for the treatment of atopic dermatitis in Japan. Delgocitinib achieved efficacy in atopic dermatitis, alopecia areata, and chronic hand eczema. Clinical trials on inverse psoriasis and discoid lupus erythematosus are ongoing.^459^ Common adverse events include mild-to-moderate nasopharyngitis (25.9%), contact dermatitis (4.5%), and acne (4.3%). Seven serious adverse events were reported, one being Kaposi’s varicelliform eruption.^460^

*Cerdulatinib*: Cerdulatinib, also known as PRT062070, inhibits JAK1, JAK2, TYK2, and SYK. Preclinical studies revealed cerdulatinib potently inhibited the proliferation of B-cell lymphoma cell lines.^461^ A phase 1 study revealed that cerdulatinib was well-tolerated and demonstrated promising antitumor effects in B-cell or T-cell non-Hodgkin lymphoma.^462^ More clinical data on cerdulatinib are needed.

### Comparisons between JAK inhibitors

As we discussed before, dozens of JAK inhibitors are used in various diseases. Thus, comparisons between JAK inhibitors are clinically meaningful.

In RA, there are six JAK inhibitors that have received market approval or are undergoing clinical trials. They are tofacitinib, baricitinib, filgotinib, upadacitinib, decernotinib, and peficitinib. For patients who are refractory to conventional RA treatment, All JAK inhibitors achieved efficacy in ACR20 (American College of Rheumatology 20% response) and DAS28 (Disease Activity Score in 28 joints). Increasing the dose of baricitinib (4 mg versus 2 mg), tofacitinib (10 mg versus 5 mg), upadacitinib (30 mg versus 15 mg) does not provide significant additional benefits.^463^ Moreover, compared to biological DMARDS, JAK inhibitors have a much shorter half-life, indicating that they are suitable for RA patients with comorbidities, such as heart diseases. For a certain index, in CRP-DAS28 (C-reactive protein) for LDA (low disease activity) and remission, upadacitinib is superior to other JAK inhibitors. In ESR-DAS28 (Erythrocyte sedimentation) for remission, tofacitinib achieved the best efficacy. For safety data, there were 11 deaths reported in tofacitinib and more serious infections in upacitinib.^464^

In IBD, all four JAKs are involved in the signal transduction of proinflammatory cytokine, and four JAK transcripts are significantly upregulated in the intestinal mucosa of patients with active ulcerative colitis.^465^ Thus, pan-JAK inhibitors may be particularly suitable for treating IBD. Various JAK inhibitors are undergoing clinical trials, including tofacitinib, filgotinib, upadacitinib, peficitinib, itacitinib, TD-1473. A systematic review compared tofacitinib, filgotinib, peficitinib, and TD-1473. Treatment with four JAK inhibitors can increase the clinical remission rate of Crohn’s disease by 38% and the clinical remission rate of ulcerative colitis by more than threefold. Similar therapeutic effects were observed in patients naive to TNF antagonists compared to patients with previous exposure, and tofacitinib ranked the highest remission in patients with previous exposure to TNF antagonists.^466,467^ For adverse events, mortality was not increased in JAK inhibitor treatment compared to placebo. Nevertheless, JAK inhibitors increase infection risk, especially herpes infection, which could be mitigated by the injection of a vaccine.^468^ There are several clinical trials completed in the past 2 years, an updated meta-analysis could be meaningful.

In alopecia areata, tofacitinib, ruxolitinib, and baricitinib are used in clinical trials. Oral JAK inhibitors were associated with four times higher odds of achieving response compared with topical JAK inhibitors, with no difference between tofacitinib, ruxolitinib, and baricitinib.^469^ More studies are needed to identify the role of JAK inhibitors in the therapy of other types of hair loss, such as Androgenetic alopecia and cicatricial alopecia.

In COVID-19, there are three JAK inhibitors undergoing phase 2/3 clinical trials, and they are tofacitinib, baricitinib, and ruxolitinib. Baricitinib and ruxolitinib were associated with a reduced risk of mortality.^470^ They reduced the use of invasive mechanical ventilation and had a borderline impact on the admission rate of the intensive care unit (ICU) and the incidence of acute respiratory distress syndrome (ARDS). Nonetheless, none of them decreased the length of hospitalization. Besides, the high cost and adverse events may limit the application of JAK inhibitors in COVID-19.^382^ More data are needed to illustrate the timing of JAK inhibitors treatment during the course of COVID-19 may affect the outcome.^471^

In atopic dermatitis, seven JAK inhibitors are undergoing clinical studies. Four (baricitinib, upadacitinib, abrocitinib, gusacitinib) were orally administered, the remaining three (tofacitinib, ruxolitinib, delgocitinib) were topically administered. A meta-analysis of 15 RCTs showed that JAK inhibitors were more effective in achieving eczema area and severity index-75 (EASI-75), Investigator’s Global Assessment (IGA), and itching-NRS responses than placebo. For the subgroup analysis, gusacitinib seems unlikely to achieve EASI-75, IGA responses, and topical delgocitinib had higher rates of achieving EASI- 75, while topical tofacitinib and ruxolitinib had higher rates of achieving IGA and pruritus-NRS. Ruxolitinib and delgocitinib have fewer TEAEs. A head-to-head meta-analysis may be essential for more data about the comparisons between JAK inhibitors in atopic dermatitis.^472,473^

### STAT inhibitors

JAK inhibitors can prevent phosphorylation and activation of STATs. However, other signaling pathways can also be inhibited. More adverse events may ensue from the inhibition of upstream tyrosine kinases. Thus, STAT inhibitors seem to be more specific with fewer adverse effects. Among all seven STATs, inhibitors targeting STAT3 and STAT5 have been the most widely studied.^474^ However, STATs do not have intrinsic catalytic activity, thus, drug research for STATs is challenging. Most studies are based on preclinical research, and few drugs are in clinical trials or market-approved because high concentrations are required for them to be effective. Most STAT inhibitors focus on restricting STAT phosphorylation and/or dimerization by peptidomimetic approaches, virtual or library screening. STAT activity can be inhibited by drugs that are not pathway-specific, such as resveratrol and curcumin. Other approaches include non-peptide small molecules and oligonucleotide-based STAT inhibitors specific to the STAT–DNA-binding domain. Antisense oligonucleotides (ASOs) interfere with STAT mRNA.

#### Peptides and peptidomimetics

The STAT3 SH2 domain is required for STAT dimerization. Thus, inhibitors targeting pTyr-SH2 interactions have been developed. The first SH2-binding peptide and peptidomimetic, named PY*LKTK (Y = phosphotyrosine), was developed in 2001. It is a phosphotyrosyl protein that binds to the native C-terminal STAT3 SH2 domain, inhibits STAT1 to a lesser extent and has no effect on STAT5. PY*LKTK and its tripeptides PY*L and AY*L disrupt STAT3:STAT3 dimerization, and blocks STAT3-mediated DNA-binding activity and gene regulation.^474,475^ In addition, no further studies of PY*LKTK were found. Peptidomimetics have better pharmacokinetic properties than peptides. With XpTL as the basic structural scaffold for developing peptidomimetic compounds, ISS 610 and S31-M2001 showed superior pharmacokinetic profiles.^476^ Through amide coupling to the Leu residue’s free acid, newly modified ISS 610 was found to have reduced selectivity against STAT3 and greater selectivity for STAT1. This new peptidomimetic is called ISS 840. ISS 840 inhibited STAT1 or STAT3 homodimerization, with 20-fold higher inhibition of STAT1 dimerization compared to STAT3 dimerization.^477^ PM-73G is a cell-permeable, phosphatase-stable phosphopeptide mimic. It targets the STAT3 SH2 domain and inhibits the phosphorylation of STAT3 at Tyr705. PM-73G exhibited antitumor efficacy in a breast cancer murine model, inhibiting VEGF production and reducing vessel density. These findings indicate the role of PM-73G as a novel anti-angiogenesis drug.^476^ Several other peptidomimetic molecules were developed from the basic scaffold of Ac-PYLPQTV-NH2, such a CJ-1383.^478^ All the aforementioned peptidomimetics need to be further studied in vivo to improve their metabolic susceptibility and cellular permeability prior to clinical applications.^479^

PIAS3 protein serves as a negative regulator of STAT3. It has been reported that ~89% of human glioblastoma samples demonstrate low expression of PIAS3 and elevated STAT3 expression, and the ectopic addition of PIAS3 to glioblastoma cells results in inhibition of pSTAT3 activity.^480^ rPP-C8, a derivate of PIAS3, is a polypeptide derived from the C-terminal acidic region of PIAS3. It is derived from the interacting domains of STAT3 and PIAS3. A protein transduction domain consisting of 9 arginine residues was added to the primary sequence of rPP-C8 to improve its cellular permeability. This modified rPP-C8 slowed cell growth and inhibited the migration of breast and brain cancer cell lines.^481^

#### Small-molecule inhibitors

##### STAT3 inhibitors

Small-molecule inhibitors constitute the largest portion of STAT inhibitors. Most of them have been identified through computational modeling, docking studies, and virtual screening of chemical libraries. Stattic (STAT Three Inhibitory Compound) was discovered by high-throughput screening of chemical libraries consisting of 17298 substances. It is the first non-peptide small-molecule inhibitor that targets STAT3. Stattic selectively inhibits the activation, dimerization, and nuclear translocation of STAT3.^482^

STA-21 (also named NSC628869) and LLL-3 are structural analogs developed by the same group. They were identified by screening the National Cancer Institute (NCI) chemical library. Both bind to STAT3 SH2 domain and inhibit STAT3 dimerization. Notably, LLL-3 has better cellular permeability than STA-21.^483^ In a glioblastoma murine model, LLL-3 inhibited STAT3 activation and tumor progression.^484^ LLL12 is generated by replacing the acetyl group of LLL-3 with sulfonamide. It specifically inhibits STAT3 phosphorylation. LLL12 combined with chemotherapy better inhibited the cell viability, cell migration, and cell growth of several ovarian cancer cell lines, such as A2780, SKOV3, CAOV-3, and OVCAR5 cells.^485^ LLL12 has also been studied in several other diseases, including non-small cell lung cancer, osteosarcoma, multiple myeloma, acute lung injury, pancreatic cancer, rhabdomyosarcoma, hepatocellular cancer, medulloblastoma, and glioblastoma. LLL12 has shown promise for use in clinical settings.^486–491^ Recently, a study showed that XZH-5 blocks STAT3 phosphorylation at Try705, and inhibition of STAT3 signaling by XZH-5 could induce the apoptosis of human breast and pancreatic cancer cells.^492^ OPB-31121 inhibited both constitutively activated and IL-6-induced STAT3 activation in gastric cancer models. Moreover, OPB-31121 showed synergistic activity with 5-FU and cisplatin.^493^ A related phase 1 clinical trial with 25 patients demonstrated the feasibility of using OPB-31121 to treat advanced solid tumors, with two patients exhibiting tumor shrinkage. Common adverse events were gastrointestinal events.^494,495^ In 2011, Turkson and his colleagues found that compounds 10, 11, and 12 inhibit STAT3-DNA-binding activity via structural-based high-throughput screening of NCI chemical libraries. S3I-201 (compound 10) is the strongest inhibitor of STAT3-DNA binding. It is effective in treating several diseases in murine models, such as autism, dry eye, liver fibrosis, obstructive nephropathy, and tumors.^496–501^

Other small-molecule inhibitors, including S3I-201.1066, S3I-1757, STX-0119, BP-1-102, OPB-11077, Napabucasin (BBI608), Pyrimethamine (GLG-801), TTI-101 (C188-9), Nitazoxanide, WP1066, and BP-5-087 have also been investigated to target STAT3. In general, the applications of STAT3 inhibitors are extensive, and more clinical data are needed to verify their safety and efficacy.

*Other STAT inhibitors (STAT1, STAT2, STAT4, STAT5,* and *STAT6)*: Compared to STAT3 inhibitors, inhibitors of STAT1, STAT2, STAT4, STAT5, and STAT6 are reported much less frequently, and most of these compounds are natural products. Few small-molecule inhibitors have been reported. However, pravastatin repressed IFNγ-mediated STAT1 activation to prevent aortic atherosclerosis. Pravastatin was developed to decrease plasma cholesterol by inhibiting 3-hydroxy-3-methylglutaryl coenzyme A reductase.^502^ Recently, pravastatin was found to repress the IL-6/STAT3 signaling pathway in rats with preeclampsia, alleviate oxidative stress and decrease the apoptosis of placental trophoblastic cells.^503^ Pimozide belongs to the diphenylbutylpiperidine class of drugs. When pimozide was used to treat schizophrenia, researchers found that the treated patients had a lower incidence of some types of cancers, including respiratory tract, prostate, and bladder cancer. Further studies have revealed that pimozide induces apoptosis and suppressed metastasis in vivo and ex vivo.^504^ Pimozide treatment reduced prolactinoma growth and increased apoptosis and cell cycle arrest in bromocriptine-resistant prolactinoma via the inhibition of the STAT5/Bcl-xL and STAT5/cyclin D1 pathway.^505^ There are several other molecules that inhibit STAT5 or STAT6, such as chromone-derived nicotinylhydrazone, BP-1108, BP-1075, leflunomide, and niflumic acid.

#### Natural products and derivatives

Natural products achieved therapeutic effects by influencing multiple biological processes, some of which inhibit the JAK/STAT pathway, and most of them target more than one component of the JAK/STAT pathway, including JAK, STAT, and inhibitory proteins. It should be noted that their effects might be indirect. Dozens of natural products are comprehensively reviewed in the previous articles, such as emodin, aloin, capsaicin, avicin D, celastrol, withaferin A, thymoquinone, caffeic acid, vinorelbine, paclitaxel, evodiamine, cryptotanshinone, honokiol, berbamine, cinnamon bark, and indirubin.^506–509^ Most of them are in the preclinical research stage, few are widely studied in clinic, we will introduce the most widely clinic studied natural products in the following.

##### Curcumin

Curcumin is a naturally occurring nutraceutical compound extensively found in the rhizome plant *Curcuma longa*. Curcumin inhibits STAT3 and induces the apoptosis of human glioblastoma and squamous carcinoma cells.^510,511^ In addition to direct inhibition of STAT3, curcumin diminishes the expression of STAT3 and STAT6 by upregulating SOCS1, SOCS3, and PIAS3.^512,513^ FLLL11, FLLL12, FLLL32, and FLLL62 are generated from curcumin and possess better biochemical properties than curcumin. These derivatives inhibit STAT3 phosphorylation at Tyr705 and induce caspase-dependent apoptosis of melanoma cells without abrogating IFNγ-induced STAT1 phosphorylation or gene expression.^514^ HCT-15 cells were co-cultured with lymphocytes from 20 stage III colon cancer patients/healthy donors. FLLL32 inhibited PD-L1 expression, decreased the number of Treg cells, and promoted Th1-protective immune responses.^515^ Besides, FLLL32 inhibited STAT3 phosphorylation induced by IFNα and IL-6 in breast cancer cells and retarded tumor growth in chicken embryo and mouse models.^516^

##### Resveratrol

Resveratrol, a natural polyphenolic stilbenoid, is found in grapes, mulberries, peanuts, rhubarb, etc. At the molecular level, resveratrol targets inflammatory cytokines, nuclear factor-κB, sirtuin, adenosine monophosphate kinase, and antioxidant enzymes.^517^ Resveratrol regulates immune responses by suppressing the phosphorylation of STAT1, STAT3, and NF-κB signaling pathways.^518^ In leukemia (Jurkat, SUP-B15, and Kasumi-1 cell lines), resveratrol inhibited IL-6-mediated STAT3 activation, induced apoptosis, and cell cycle arrest. Resveratrol also prolonged the survival period of tumor-bearing mice.^519^ However, resveratrol has poor bioavailability. LTR71 (6-methyl-2-propylimino-6, 7-dihydro-5H-benzo[1,3]-oxathiol-4-one), a derivative of resveratrol, suppressed RANTS induced STAT3 activation in breast cancer cells. Moreover, LTR71 inhibited the expression and activity of MMP-9 and prolonged the survival of murine models.^520^ Furthermore, the substitution of hydroxyl groups with methoxy groups improved the therapeutic versatility of resveratrol, and relative derivatives include pterostilbene, trimethoxystilbene, tetramethoxystilbene, and pentamethoxystilbene. In another method, 4-hydroxy group in the trans conformation is added to the 4- and 4’-positions in the stilbene structure. These derivatives include dihydroxystilbene, tetrahydroxystilbene, and hexahydroxystilbene. Piceatannol is a hydroxylated derivative that shows similar functions to resveratrol. It is regarded as a promising drug in treating aging-related diseases and various cancers.^521^ The third group of resveratrol derivatives is generated from the addition of halogen groups to the stilbene structure, such as 2-bromo-resveratrol and 2-chloro-resveratrol.^522^

##### Oleanolic acid

Oleanolic acid, a pentacyclic triterpenoid compound, exists in over 2000 plant species, especially the Oleaceae family. It is reported that oleanolic acid interfered with TYK2-STAT1/3 signaling, significantly decreased STAT3 dimers formation, and promoted the expression of SOCS3.^523,524^ To promote the clinical application, oleanolic acid can be structurally modified on the A ring, c-28, and C ring. Its derivatives include novel 3,5-disubstituted isoxazoles derivatives, acyloxyimino derivatives, 3-acetylated derivatives, acetate, ester derivatives, and oximes derivatives. Bardoxolone is one of the most widely studied derivatives. Moreover, nanometer preparation could improve the biochemical properties of oleanolic acid.^525^

##### Catechins

Catechins, also named flavanes, belong to the class of flavonoids. Catechins are the major constituent of green tea. Catechins are well-known for their antioxidant, anti-inflammation, and anticancer effects. Catechins affect multiple signal pathways, such as JAK/STAT, MAPK, PI3K/AKT, Wnt, and Notch.^526^ Catechins exhibited the chemoprotective effect during cancer therapy, partly due to inhibiting STAT1 and STAT3, then decreasing the expression of iNOS and ICAM-1.^527^

##### Artemisinins

Artemisinins are extracted from the sweet worm-wood (*Artemisia annua*). Artemisinin is used to treat fever in Chinese history, and it is famous for treating malaria. Besides, artemisinins also have anticancer, anti-virus, anti-schistosomes, anti-inflammatory properties, and artemisinins are effective in ocular diseases.^528–531^ Derivatives of artemisinin are collectively termed artemisinins.^532^ In preadipocytes and cancer cells, artemisinins interfered with STAT3 dimerization by binding to the SH2 domain, then inhibited STAT3 target gene expression.^533,534^

##### Clinical application and limitations of natural products

There are many clinical trials that have been completed or are in progress. For example, clinical studies on Curcumin are more than 250 on www.clinicaltrials.gov. Besides, their application covers a wide range of diseases. We will briefly introduce the clinical applications of curcumin, resveratrol, oleanolic acid, catechins, and artemisinins in the following.

Clinical trials of curcumin cover cancers, oral diseases, autoimmune diseases, metabolic diseases, cardiovascular diseases, neurological diseases, such as oral submucous fibrosis, periodontitis, osteoarthritis, nonalcoholic fatty liver disease, diabetes, IBD, polycystic ovary syndrome, radiation dermatitis, migraine, psychiatric disorders, and COVID-19. Most of them reported positive results, indicating that curcumin was well-tolerated and biologically active. Adjuvant therapy of curcumin may reduce fatigue in cancer patients.^535^ Curcumin and its derivatives can produce various dermatological effects, such as antioxidant, anti-inflammatory, reducing axillary hair growth, and increasing skin moisture. Further studies could explore the application of curcumin in dermatological diseases.^536^

Clinical trials regarding resveratrol achieved benefits in many human diseases, such as diabetes, obesity, cardiovascular diseases, neurodegeneration, muscular dystrophies, and cancers. Notably, resveratrol contributes to improve physical functions. For example, resveratrol and curcumin promote the recovery of bone and muscle mass in chronic kidney disease patients.^537^ Resveratrol combined with exercise in the elderly could improve physical functions and mitochondrial function.^538^

In clinical trials, oleanolic acid is studied in metabolic diseases including diabetes, obesity, and hyperlipidemia. Based on the published data, Oleanolic acid has anti-inflammatory, anticancer, antiosteoporotic, antioxidant, antiaging, neuroprotective, and hepatoprotective effects.^539^ Based on the preclinical data, future clinical studies should focus more on cancer, neurological diseases, cardiovascular diseases, oral diseases, and hepatitis.

Clinical trials of catechins revealed their therapeutic efficacy in diabetes, hyperlipidemia, hypertension, and obesity. Several clinical studies focus on multiple sclerosis, hepatitis, acute radiation-induced esophagitis. Notably, catechin may have regional analgesic efficacy for pain relief after surgery.^540^ Besides, Catechin combined with xanthan gum protect against upper respiratory infections.^541^

The clinical application of artemisinins is mainly for malaria. In the past 10 years, there have been few reports of clinical trials of artemisinin in the treatment of schistosomiasis. A few clinical trials have revealed that artemisinins are well-tolerated in cancer patients. A larger scale of clinical trials in cancer or metabolic diseases is needed to identify the optimal dose and efficacy of artemisinins.

Nevertheless, there are multiple limitations of natural products. First, many of them simultaneously destroy abnormally proliferating cancer cells and normal cells, partly due to the broad-spectrum inhibition of biological processes. Second, many natural products are underutilized owing to intrinsic pharmacokinetics, including short half-life, low bioavailability, inadequate biological stability, and poor aqueous solubility. Third, some natural products have hepatotoxicity, kidney toxicity, and reproductive toxicity. New developments aim to overcome these obstacles, such as synthesizing derivatives of natural products and utilizing nanoparticles, exosomes, liposomes, and phospholipids.

#### Nucleotide-based agents

##### Decoy oligonucleotides

Oligodeoxynucleotide (ODN) decoys are specific DNA-binding domain inhibitors. They compete with endogenous promotor sequences for binding to active STAT-STAT dimers, thus suppressing gene expression.^506^ For example, the STAT1 decoy ODN effectively inhibited murine antigen-induced arthritis and acute rejection of mouse/rat heart allografts, and as verified by in vitro study, nuclear extracts from synoviocytes were inhibited by STAT1 decoy ODN, as determined through electrophoretic mobility shift analysis.^542–544^ Decoy ODN targets both STAT1 and STAT3 to decrease allergic inflammation in rat asthma.^545^ In summary, most decoy ODNs targeting STAT are in the preclinical investigation stage of development. More studies are needed to further promote their application.

##### Antisense oligonucleotide

ASO targets a complementary coding sequence of mRNA and downregulates STAT expression at the transcriptional level. ASO is used in the inhibition of STAT and blocks cells from malignant transformation. Limitations of ASO include elevated liver enzymes, splenomegaly, and thrombocytopenia.^546,547^ AZD9150, a STAT3 ASO, was evaluated in diffuse large B-cell lymphoma patients and was well-tolerated and effective.^548^

##### STAT-targeting small interfering RNAs (siRNAs)

siRNAs are RNA duplexes of 19–22 bp. They incorporate their guide strands into a structure named the RNA-induced silencing complex. This process naturally occurs and is termed RNA interference.^549^ For example, STAT5B expression that was blocked by siRNA enhanced the chemosensitivity of gastric cancer cells, contributing to increased mitochondrial pathway-mediated apoptosis.^550^

Peripheral neuropathy and increased mortality are toxicities of concern in siRNA therapy. New modifications enable lower dosing and exposure levels while maintaining efficacy and will hopefully show fewer adverse effects.^549^

### New developments and challenges of JAK/STAT inhibition

Some JAK inhibitors are newly developed and/or rarely reported, including pan-JAK inhibitor: TD-1473; JAK1 inhibitor: AZD4205, INCB-047986, INCB039110, INCB-052793; JAK2 inhibitor: AZD1480, AC-410; JAK3 inhibitor: Ritlecitinib (PF-06651600); TYK2 inhibitor: Deucravacitinib (BMS-986165), brepocitinib (PF-06700841), PF-06826647.^93,551–553^ These newly developed JAK inhibitors are expected to have increased selectivity and reduced adverse events. For example, TD-1473 is a gut-selective pan-JAK inhibitor used in ulcerative colitis. The novelty of TD-1473 is that it can locally inhibit proinflammatory cytokines in the gastrointestinal tract while minimizing systematic exposure.^553^ Moreover, multi-kinase inhibitors may achieve unexpected effects. Gusacitinib and cerdulatinib inhibit JAK and SYK, both JAK and SYK are involved in the pathogenesis of atopic dermatitis. Newly developed ritlecitinib inhibits JAK3 and the tyrosine kinase expressed in hepatocellular carcinoma kinase family, and ritlecitinib has achieved efficacy in RA patients.^554^

There are still many unanswered questions and challenges in the clinical application of JAK inhibition. First, optimizing dosing and formulation is important. For short half-lives JAK/STAT inhibitors, pulsatile high doses may result in more effective targeting, fewer adverse effects, and less resistance.^555^ Besides, similar to steroids, patients could be given a bolus dose of JAK/STAT inhibitor to induce remission and gradually reduce the dose for maintenance. Second, in clinical trials, JAK/STAT inhibitors elevated physical signs and symptoms in many autoimmune diseases. Nevertheless, rare significant anticancer effects are observed except for hematological tumors. This is partly due to the complex immune microenvironment in cancer patients. JAK/STAT pathway can both promote tumor progression and mediate anticancer effects. Moreover, JAK/STAT inhibitors may inhibit the function of cytotoxic T cells and NK cells.^556^ Cross-talk between JAK/STAT pathway and other pathways, post-translational control, epigenetic modification, and noncanonical signal transduction make the mechanisms more complicated. Third, for patients with liver impairment, renal impairment, risk of thromboembolism, and chronic virus infection, such as HBV, the dose should be reduced according to the condition.^365,366,447,448^ Forth, although most adverse events are well-tolerated, there is a possibility of life-threatening adverse events, such as severe infections and malignancies, ruxolitinib discontinuation syndrome, and Wernicke’s encephalopathy.^403,406,407,431^ Fifth, combinational therapy may provide additional benefits. For example, hsp90 inhibitors or histone deacetylase inhibitors promote JAK2 degradation.^557^ In addition, it is reported that many JAK inhibitors, such as baricitinib and fedratinib, downregulated the PD-L1 expression. Thus, combinational therapy of JAK inhibitor and immune checkpoint blockers may augment therapeutic efficacy.^556^

The most important limitation of STAT inhibitors is their toxicity, which comes from several aspects. First, there is considerable homology between different STATs, thus it is difficult to design highly specific STAT inhibitors, which can lead to off-target toxicity. Second, STATs participate in biological processes in the mitochondrion and endoplasmic reticulum. Therefore, STAT inhibitors may interfere with these processes.^142^ Third, the function of a certain STAT could be compensated by other STATs. For example, STAT3-deficient cells still respond to IL-6 stimulation through STAT1 transduction.^5^

## Conclusion and future directions

The JAK/STAT pathway is central to extracellular cytokine-activated receptor-mediated signal transduction, which is involved in cellular proliferation and differentiation, organ development, and immune homeostasis. In this review, we discussed the composition and function of the JAK/STAT pathway and discussed the role of JAK/STAT in various diseases. Dysregulation of the JAK/STAT signaling pathway is recognized as a major contributor to various diseases, especially malignant tumors, and autoimmune diseases.

Diseases characterized by hyperactivated JAK/STAT pathway, elevated serum JAK-dependent cytokines, and mutated JAK/STAT are thought to respond well to JAK/STAT inhibitors. JAK/STAT inhibitors are currently applied in autoimmune diseases, malignant tumors, GVHD, and infectious diseases. Research into more clinical indications is ongoing, including IL-6 driven diseases: large-vessel vasculitis, type I IFN-related diseases: monogenic interferonopathies, scleroderma, myositis, and primary Sjogren’s syndrome.

There are still some unanswered questions about signal transduction. First, although JAK/STAT signal transduction is usually presented in a simple way, decades of research have shown that it is full of complexity. For example, the functions of STATs in organelles need further to be studied. In addition, JAKs and STATs are regulators of the epigenetic landscape, conversely, they are regulated by the epigenetic landscape, via promoting permissive marks and limiting repressive marks. For instance, JAK2 phosphorylates histone H3 at tyrosine 41, thus decrease the affinity of H3 to HP1α, promote tumorigenesis.^557^ Second, STAT can directly bind to DNA, but where and how they bind is an ongoing issue of debate, deep sequencing and chromatin immunoprecipitation (ChIP-seq) may help construct a comprehensive and unbiased STAT–DNA-binding map. Third, more research is needed to explain how STAT-mediated transcriptional inhibition works, either through direct binding of genomic locales or through the induction of secondary agents, such as inhibitory transcription factors and miRNA. Fourth, STATs can be activated by different cytokines, conversely, a cytokine can activate multiple STATs. Different cytokines are seen as different signals, a putative explanation is that different cytokines activate different phosphorylation levels of various STAT and other signal modules. More studies are needed to support the hypothesis. Fifth, how JAK/STAT pathway participates in the pathogenesis of diseases is not fully elucidated. For example, in the case of JAK2^V617F^mutation of MPN, how does the JAK/STAT pathway go wrong?^558^ Sixth, most diseases result from multiple genetic abnormities, the cross-talk between JAK/STAT pathway components and other pathway components has not been fully elucidated.

Future studies should offer transformative insights into the underlying mechanisms of the JAK/STAT pathway effects and disease development. Moreover, we should aim to maximize efficacy and minimize adverse effects in patients in different stages of certain diseases and to explore biomarkers that predict efficacy and offer prognoses.
